# Understanding the hydride precipitation mechanism in HCP Zr polycrystals: a micromechanical approach

**DOI:** 10.1007/s10853-025-10796-8

**Published:** 2025-04-10

**Authors:** Yang Liu, Mark R. Wenman, Catrin M. Davies, Fionn P. E. Dunne

**Affiliations:** 1 Department of Materials, Imperial College London, London, SW7 2AZ UK; 2 Department of Mechanical Engineering, Imperial College London, London, SW7 2AZ UK; 3 School of Engineering, University of Leicester, Leicester, LE1 7RH UK

## Abstract

This study focuses on the hydride precipitation in zirconium polycrystals during thermo-mechanical cycles. The precipitation and dissolution of mesoscale hydrides in Zircaloy-4 is modelled using crystal plasticity finite element methods supported with DFT-informed zirconium lattice hydrogen concentration. Results for a tri-crystal case show the effects of crystallography, thermo-mechanical load and elasto-plastic anisotropy on hydride nucleation and growth. Analyses of polycrystalline models provide new insights into the complex precipitation process of hydrides in Zircaloy-4 with explicit representation of experimental observations that lay the foundation for further research in this field. Micromechanical findings demonstrate the importance of microstructure, pre-thermal condition, and hydrogen concentration limit on hydride precipitation. Overall, the study provides a deeper understanding of hydride formation during industrially relevant reactor conditions.

**Graphical abstract:**

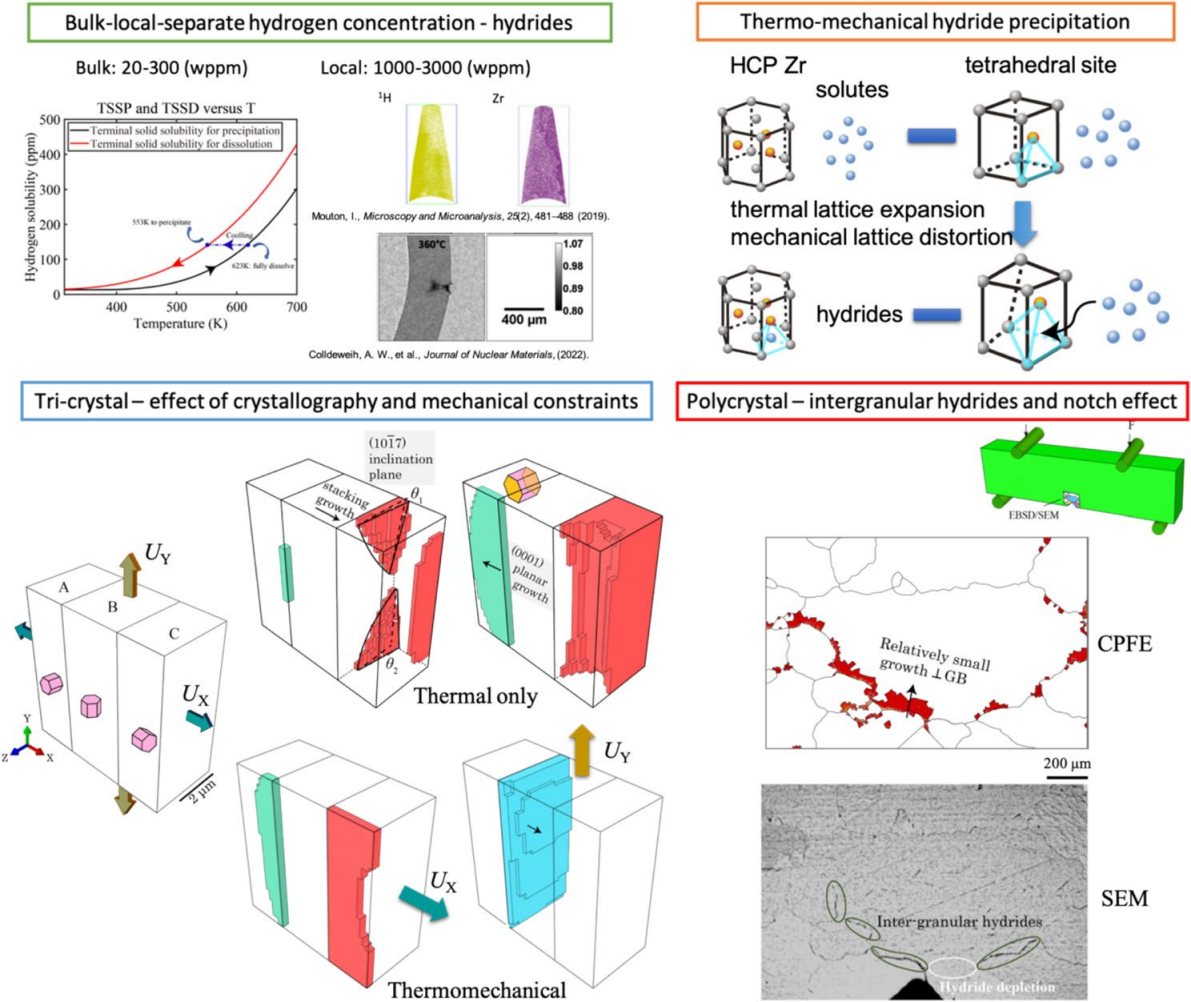

## Introduction

Zircaloy-4 is widely used as fuel cladding material in the nuclear industry due to its unique neutron transparency, combination of mechanical strength, thermal stability, and corrosion resistance. However, its usage is associated with the formation of hydrides since cladding is immersed in water at temperature up to ~ 620 K, which accelerates the oxidation process producing hydrogen, which induces hydrogen pick-up. Due to drastic drop of hydrogen solubility from 620 K to room temperature [1, 2], hydride precipitates within the cladding, leading to hydrogen embrittlement, fracture toughness reduction, which can significantly impact the overall performance of nuclear cladding, and in some cases a time-dependent failure mode called delayed hydride cracking (DHC) [3]. The study of the mechanism of hydride precipitation in HCP alloys has gained serious attention in recent years [4–9], as it has a profound impact on the safety and the economics of nuclear reactors. However, the mechanisms behind hydride precipitation and DHC have not been fully understood.

At the microstructure scale, the packets of microhydrides that form into macrohydrides dominate the structural integrity of zirconium cladding. Hydride packets follow a morphological habit plane to {10$\overline{1 }$7}_α_ of α-Zr and contains hydride platelets with habit plane to {0002}_α_ [10]. δ hydride (ZrH_1.6_) is the most observed hydride phase with FCC atomic structure [11], and two hydride-matrix (α-δ) ORs have been observed [12], i.e. OR1 ${\left\{ {0001} \right\}_\alpha }\left\| {{{\left\{ {111} \right\}}_\delta }{{\left\langle {11\bar 20} \right\rangle }_\alpha }} \right\|{\left\langle {110} \right\rangle _\delta }$ and OR2 $\left\{ {0001} \right\}_{{\upalpha }} \left| {\left| {\left\{ {001} \right\}_{{\updelta }} \left\langle {11\overline{2}0} \right\rangle_{{\upalpha }} } \right|} \right|\left\langle {110} \right\rangle_{{\updelta }}$. OR1 was most reported. Hydride packet growth within a zirconium microstructure depends majorly on the matrix grain size and cooling rate [13, 14]. Under thermo-mechanical loads, plastic deformation occurs in the material, and associated dislocation structure evolution has been demonstrated to deeply connect with hydride dissolution and precipitation process [15–18]. These studies also conclude that when hydride dissolves, residual dislocations remain that can provide initiation sites for subsequent hydride growth. Existing δ hydride packets in zirconium alloys have a strong interaction with the α-Zr matrix, observed from micropillar compression and HRDIC testing [19, 20] and crystal-level hydride properties were investigated by coupling CP with HRDIC [21]. Gaps still exist in the understanding of the mechanism of hydride precipitation, where mechanistic understanding does not include the existing knowledge of elasto-plastic hydride packets and their interaction with the matrix, which are crucial to the process of hydride precipitation and to the structural integrity assessment during DHC.

**Figure 1:**
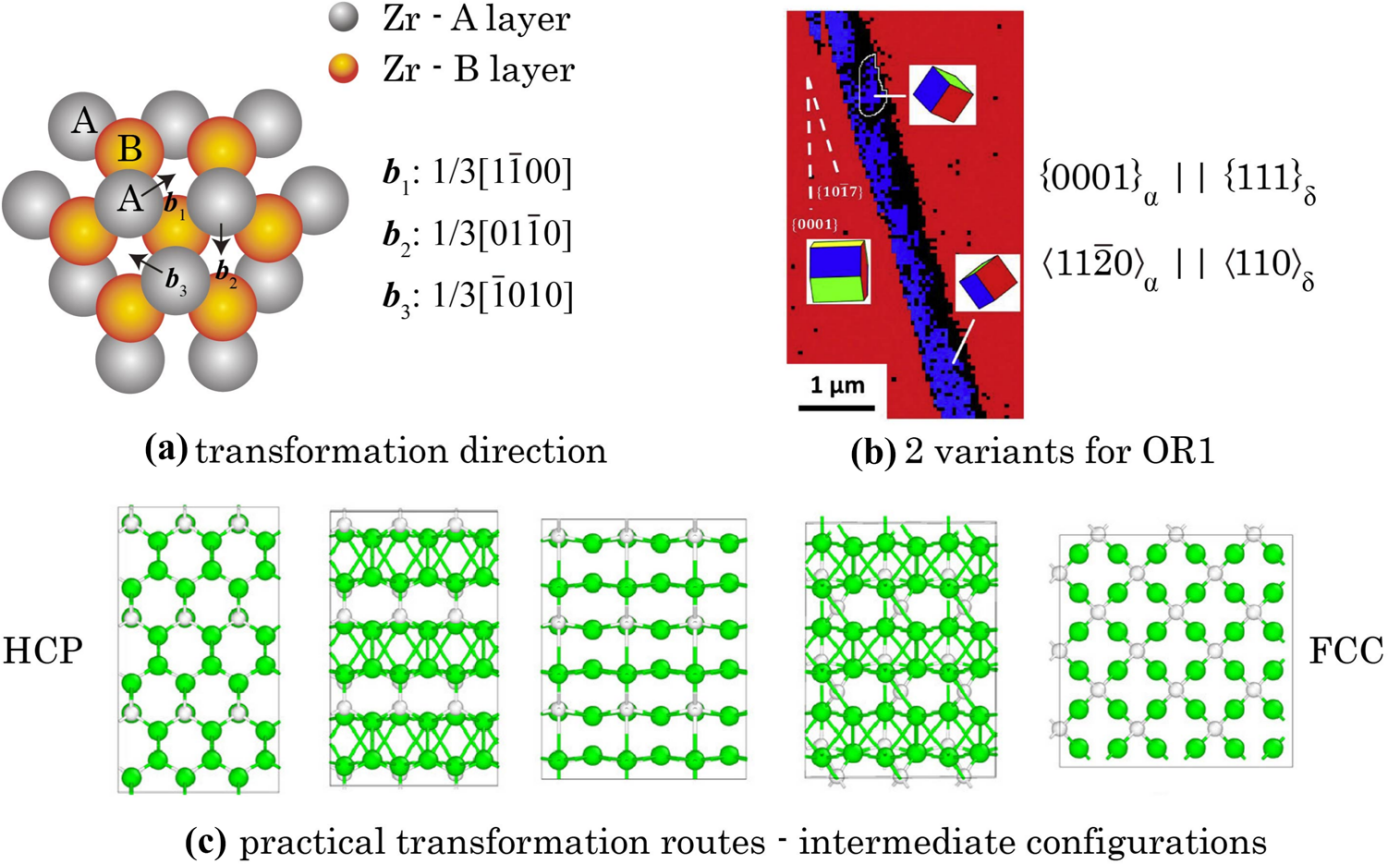
Modelling perspectives for hydride precipitation. **a** Phase field modelling considered direct HCP→FCC transition for three variants, whereas **b** two variants were observed from EBSD scan [29]. Intermediate configurations were demonstrated by molecular dynamics [4], considering interaction between H and Zr atoms.

To improve insight into this complex phenomenon, various methods have been developed to investigate the science behind hydride precipitation, which include phase field methods [22–24], hydrogen diffusion modelling [25], microstructure modelling [26–28] and experimental characterisations [12, 13, 29]. A challenge for exploring the mechanisms arises from the coupling of microstructural heterogeneity, temperature dependence and the complex interplay between hydride and metal microstructure. Polycrystalline microstructure was studied with explicit hydrides and pre-crack, which showed the importance of orientation relationships for local deformation and fracture behaviour [26]. But only intragranular hydrides were investigated, and the effect of hydride morphology and its OR with parent grain crystallography were not considered. It was reported that Han et al. [22] and Heo et al. [23] assumed three variants for OR1 in their δ hydride precipitation model, which is based on the threefold symmetry and the necessity of shearing through three $[10\overline{1 }0]$ directions for the HCP→FCC transition. This was proposed for intermetallic precipitation of γ-TiAl [30, 31], as shown in Fig. 1a. However, in experimental work, only two possible crystallographic variants were observed for OR1 within a δ hydride blister [12], the formations of which were driven by two twin variants [29], as shown in Fig. 1b.

Recent DFT calculations showed that the HCP→FCC transition for δ hydride was driven by the diffusion of hydrogen atoms and an intermediate configuration $\varsigma -Pn\overline{3 }m$ [4] as shown in Fig. 1c. This means that the phase transformation from HCP to FCC during hydride precipitation is not a single route from lattice shearing, but a complex route based on the stability of Zr–H atomic configurations, linking to the local hydrogen concentration or atomic percentage hydrogen inside the lattice cell. Furthermore, the time scale <  ~ 0.1 ps and length scale <  ~ 1 nm^3^ in former modelling methods are small [22–24], whereas structural integrity of polycrystalline materials majorly depends on the microstructure length scale of ~ µm or mm and industrially relevant timescales of hours.

This research aims to delve deeper into the mechanistic basis behind hydride precipitation in Zircaloy-4. To account for practical time and length scales for hydride evolution within Zircaloy-4, DFT-informed CPFE methods are proposed to model the nucleation and growth of hydrides in Zircaloy-4 during thermo-mechanical loads. The model incorporates explicitly the spatially resolved crystal-level properties of hydride and matrix, which provides new insights into the hydride precipitation process. The findings of this study have fundamental implications for understanding the hydride growth at microstructure scale, which presents a computational tool to improve the performance and safety of nuclear reactors.

## Methods

### Crystal plasticity theory

Explicit modelling of the most observed δ hydride precipitation is proposed for hydrided Zircaloy-4 under thermo-mechanical conditions. Thermal and elasto-plastic rate-dependent properties of δ hydride and parent α-Zr are incorporated. Slip activation occurs inside the hydride during precipitation and the material properties of δ and α were extracted from the analysis of CPFE modelling compared to HRDIC measurements of time-dependent slip evolution in hydride and local creep accumulation in blocky α grains [21, 32], respectively. These crystal-level properties are introduced in Appendix A.

In the CPFE method, the total deformation gradient is multiplicatively decomposed into elastic ***F***^e^, plastic ***F***^p^ and thermal ***F***^θ^ parts as

1 $${\varvec{F}} = {\varvec{F}}^{{\text{e}}} {\varvec{F}}^{{\text{p}}} {\varvec{F}}^{{\uptheta }}$$

***F***^θ^, introduced for Zircaloy-4 [33], represents the anisotropic thermal expansion in HCP (α) lattice and also the isotropic expansion in FCC (δ) lattice. Plastic velocity gradient ***L***^p^ is linked to the local shear strain rate, i.e. dislocation slip rate, in each slip system,

2 $${\varvec{L}}^{{\text{p}}} = \dot{\user2{F}}^{{\text{p}}} {\varvec{F}}^{{{\text{p}} - 1}} = \mathop \sum \limits_{i = 1}^{N} \dot{\gamma }^{i} {\varvec{s}}^{i} \oplus {\varvec{n}}^{i} = \mathop \sum \limits_{i} \rho_{m} b^{i2} \nu \exp \left( { - \frac{\Delta F}{{kT}}} \right)\sinh \left( {\frac{{\left( {\tau^{i} - \tau_{c}^{i} } \right)\Delta V}}{kT}} \right){\varvec{s}}^{i} \oplus {\varvec{n}}^{i}$$

where *γ*^*i*^ is the dislocation slip in *i*^th^ slip system for both HCP (*N* = 30) and FCC (*N* = 12) crystals, which activates only when RSS *τ*^*i*^ exceeds CRSS ${\tau }_{\text{c}}^{i}$; ***s*** and ***n*** are the unit vectors of slip direction and slip normal, respectively. Previous studies showed that the hyperbolic function (sinh) derived from Dunne et al. [34] was sufficient to describe the strain localisation, rate-sensitive slip activation, and irradiation-induced slip within zirconium alloys [21, 32, 35]. Thermal activation energy Δ*F*, thermal activation volume Δ*V* and CRSS *τ*_c_ are the key material properties representing the grain-level or microscale SRS [32]. *ρ*_m_ is the initial mobile dislocation density; *b* is the magnitude of the Burgers vector; *ν* is the dislocation vibration frequency. The hyperbolic sine function (sinh) has been proven to be resilient in capturing SRS across low to high strain rates [36]. Based on Taylor hardening, CRSS evolution is linked to the dislocation pile-ups of SSD and GND, i.e. increase of *ρ*_SSD_ and *ρ*_GND_,

3 $$\tau_{{\text{c}}}^{{\text{i}}} = \tau_{{{\text{c}},0}}^{{\text{i}}} + Gb^{{\text{i}}} \sqrt {\rho_{{{\text{GND}}}} + \rho_{{{\text{SSD}}}} }$$

where *τ*_c,0_ is the initial strength of dislocation slip. As forward and backward events are both accounted in the slip activation [34] in Eq. (2), the GND pile-ups near grain boundaries are expected to contribute to the long-range back stress. Local *ρ*_GND_ is derived from the Nye’s tensor ***Λ***, accounting for the lattice curvature as a function of plastic deformation gradient [34, 37, 38].

4 $$\mathop \sum \limits_{i} {\varvec{b}}^{i} \otimes {\varvec{\rho}}_{{{\text{GND}}}}^{i} = {\text{curl}}\left( {{\varvec{F}}^{{\text{p}}} } \right) ={\varvec{\varLambda}}$$

Stored energy density *G*_SED_ [39] reflects the part of the plastic dissipation that is stored in dislocation structures, leading to the crack nucleation. It is given by

5 $$G_{{{\text{SED}}}} = \frac{{U\Delta V_{s} }}{{\Delta A_{s} }} = \mathop \int \limits_{0}^{t} \frac{{\xi \left| {\sigma :D^{p} } \right|}}{{\sqrt {\rho _{{{\text{SSD}}}} + \rho _{{{\text{GND}}}} } }}~{\text{d}}t$$

where ${\text{U}}\, = \,\int \left| {\sigma :{\varvec{D}}^{{\text{p}}} } \right|$ d*t* is the dissipated energy and ***D***^p^ is the plastic deformation rate tensor. *ξ* is the fraction of plastic energy not lost as dissipated heat, but stored through the creation of dislocation structures, which is taken to be 0.05 [39–41]. Δ*V*_s_ = *λ⋅*Δ*A*_s_ is the dislocation storage volume, and Δ*A*_s_ is the potential free surface area. *λ* = 1/$\sqrt{{\rho }_{\text{SSD}}+{\rho }_{\text{GND}}}$ is the resultant mean free path of SSD and GND. Stored energy density *G*_SED_ was proposed to identify fatigue crack nucleation sites [39] and was indicative of twin nucleation sites in polycrystalline HCP materials [42].

### Hydride evolution

**Figure 2:**
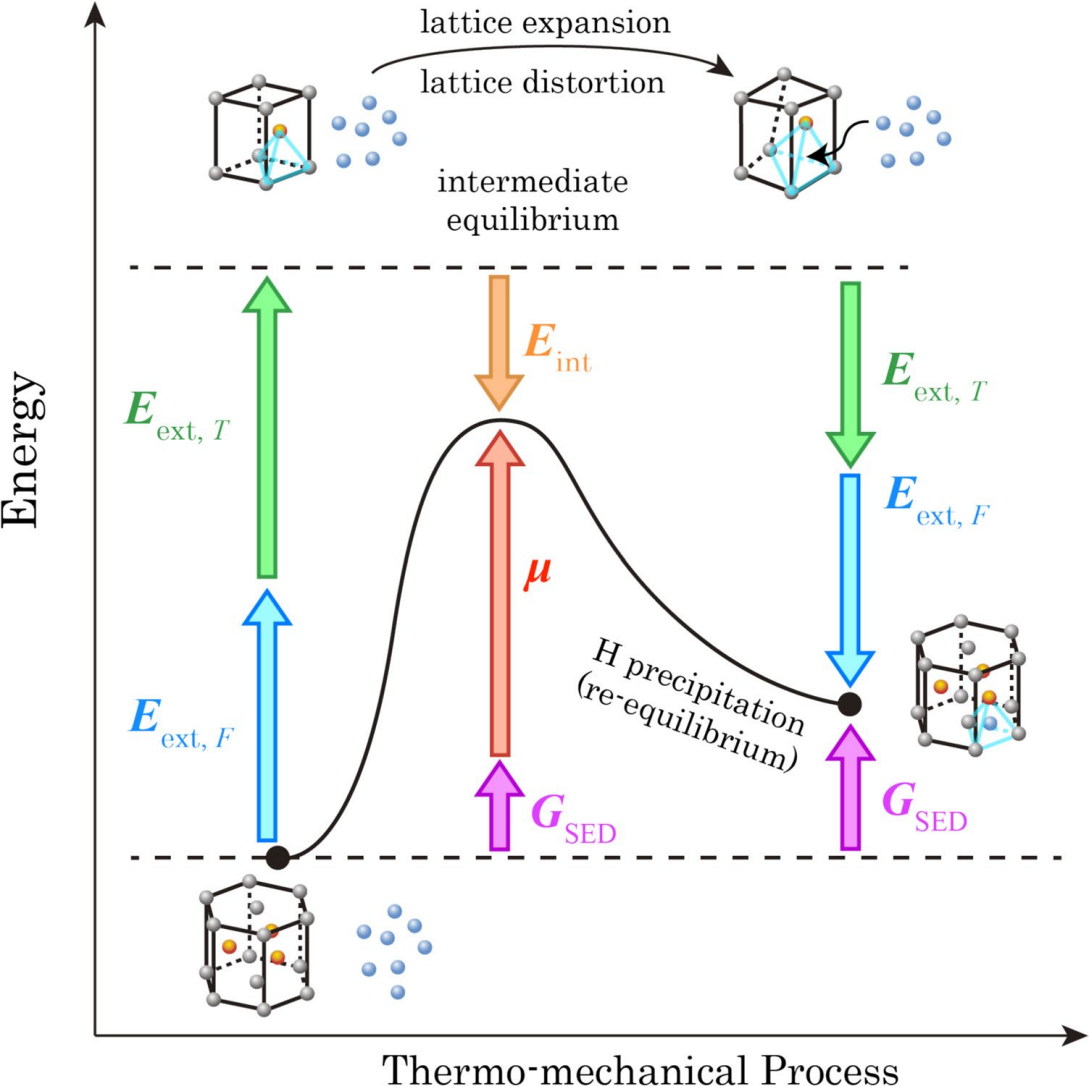
Energy evolution for hydride precipitation in thermo-mechanical process, including intermediate balance between interaction energy E_int_ and chemical potential µ, and increase of G_SED_ during hydriding.

Under the thermo-mechanical load, the energy evolution during hydride precipitation process is schematically illustrated in Fig. 2. Considering separate hydrogen and zirconium atoms at the beginning, while in-service performance kicks in, external thermal energy *E*_ext,T_ and mechanical energy* E*_ext,F_ exert onto the atomic lattice structure, leading to the expansion and distortion of α-Zr lattice, which drives the solute-state hydrogen atoms to diffuse into the preferential tetrahedral sites in zirconium lattices. Since the hydrogen diffusion coefficient is high in zirconium alloys, redistribution of hydrogen quickly stabilises and internal intermediate equilibrium is achieved between atomic interaction energy* E*_int_ and chemical potential, *µ*. Energy* G*_SED_ stored in dislocation structure also increases. When maintenance demands in fission plants, thermo-mechanical load is removed, leading to hydride precipitation due to the low solubility of hydrogen in zirconium alloy at room temperature. In the meantime, energy* G*_SED_ increases, caused by the extra dislocation accumulation from both stress concentration at the hydride-matrix boundary and the dislocation slip within hydrides. Stress concentration at the phase boundary is caused by the mismatch strain between hydride and matrix, which re-equilibrates the neighbouring hydrogen during hydride growth [43–46].

**Figure 3:**
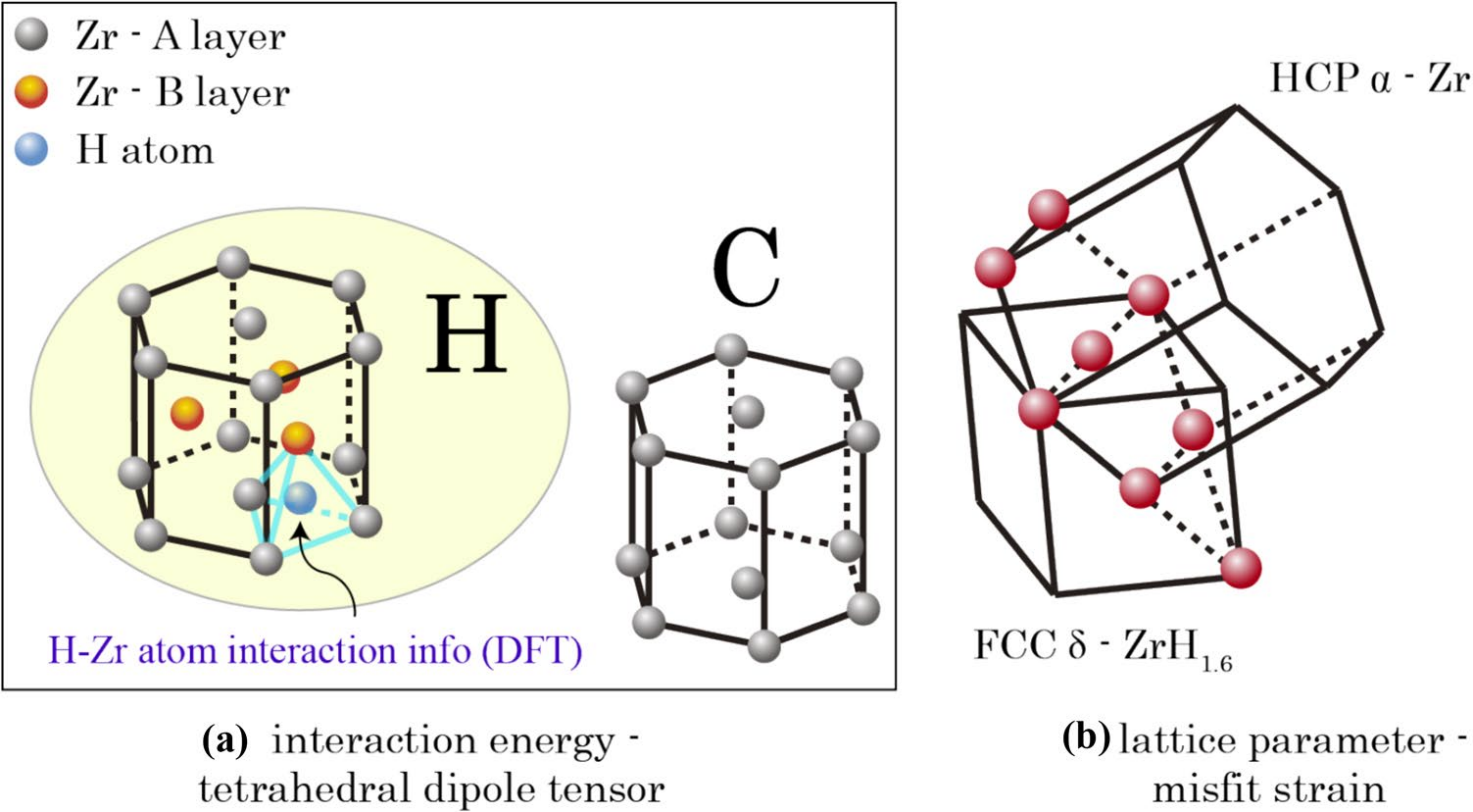
Hydride precipitation model based on **a** DFT-based atomistic information for interaction energy *E*_int_; and **b** lattice mismatch between HCP and FCC during hydride formation.

At the microstructure scale, hydride evolution depends on the heterogeneous hydrogen concentration. During thermo-mechanical loading, hydrogen distributes heterogeneously and is driven by local non-uniform hydrostatic stress resulting from elastic anisotropy, grain/phase interactions and geometrical stress raisers, e.g. notches [25, 33, 45]. At the solute level, hydrogen atoms are driven by the crystal-level stress/strain and the atomic lattice distortion caused by the interaction between hydrogen atoms and their neighbouring cells. Hydrogen atoms tend to locate in the stable tetrahedral site within the HCP α lattice shown in Fig. 3a. Local hydrogen concentration is computed based on the interaction energy *E*_int_, representing interaction between hydrogen embedded lattice cell and its surrounding lattices. An updated derivation of the hydrogen concentration based on interaction energy is introduced in the Appendix B, following our previous work [33].

Previous transient studies on hydrogen redistribution showed a quick stabilisation time constant compared with the low deformation rate [47–49]. Thus, the transients (as opposed to the multiple steady-state distributions developing as stress states change) associated with hydrogen diffusion were proven to be negligible for cooling rate <  ~ 1 K/min, which is the industrially relevant condition for nuclear fuel cladding [33].

Misfit strain from the lattice mismatch between HCP and FCC crystals is accounted for the CP during phase transformation from α to δ and the reverse, as shown in Fig. 3b, which follows the OR1 $\left\{ {0001} \right\}_{{\upalpha }} \left| {\left| {\left\{ {111} \right\}_{{\updelta }} \left\langle {11\overline{2}0} \right\rangle_{{\upalpha }} } \right|} \right|\left\langle {110} \right\rangle_{{\updelta }}$. Volumetric misfit strain ${\varvec{\varepsilon}}^{{{\text{VM}}}} = {\varvec{R}}^{{\text{T}}} \cdot {\varvec{\varepsilon}}_{0}^{{{\text{VM}}}} \cdot {\varvec{R}}$ is incorporated to accommodate the lattice mismatch during phase transformation from HCP (α) to FCC (δ) crystal structures, and ***R*** is the grain orientation matrix from EBSD scanning. ${\varvec{\varepsilon}}_{0}^{{{\text{VM}}}}$ is the volumetric misfit strain for the initial configuration [16, 25],

6 $${\varvec{\varepsilon}}_{0}^{{{\text{VM}}}} = \left[ {\begin{array}{*{20}c} {0.0458} & 0 & 0 \\ 0 & {0.0458} & 0 \\ 0 & 0 & {0.072} \\ \end{array} } \right]$$

*c*-axis misfit strain is higher due to the differing c/a lattice parameters and the higher elastic stiffness along the c-direction in the HCP crystal. However, direct addition of the volumetric misfit strain causes severe convergence problems while locally transforming the α-Zr properties to those for the newly developed δ hydride. A physically based computational method is introduced below to smooth the misfit strain transition and to resolve the convergence problem.

At the microstructure scale, hydride precipitation or dissolution is expected to happen while local hydrogen concentration exceeds or falls below a certain hydrogen concentration limit. However, hydrogen concentration limits are measured differently for Zircaloy-4 while using techniques across different scales. The bulk hydrogen concentration limits in TSSD/TSSP are the averaged hydrogen levels measured from whole samples. However, hydrogen concentration varies enormously throughout the microstructure, where fierce concentrations can arise due to e.g. constraint effects generated at boundaries and interfaces resulting from elastic anisotropy. Fundamentally, the formation of hydrides at atomic scale in metals follows the stoichiometric value of hydrogen concentration in the Zr atomic cell which is 18,200 wppm for δ hydride [50, 51]. Bulk hydrogen measurements of Zircaloy-4 using hot gas/vacuum extraction or IGF give hydrogen concentration up to 1000 wppm [33, 52–56] for high burn-up fuel rods, and the bulk hydrogen concentration from IGF for current samples is just ~ 130 wppm [33]. However, by using neutron diffraction measurement in Zircaloy-4, the local hydrogen concentration has been shown to reach up to ~ 1500 wppm near stress-raiser liner (a low-tin Zircaloy-4 layer) with bulk concentration of only 200 wppm [55]. Regarding samples with similar bulk hydrogen content and a stress-raiser crack tip (without liner), neutron diffraction showed local hydrogen concentration of ~ 1000 and up to ~ 3000 wppm near crack tip [54]. By using APT, where samples are only 10-100 s of nm in dimensions, hydrogen concentration in the α-Zr matrix adjacent to the hydride reaches up to ~ 2000 wppm with nominal bulk hydrogen concentration of only ~ 400 wppm, with maximum concentration also up to ~ 3000 wppm [57, 58]. Clearly, an order of magnitude difference exists between bulk and local hydrogen measurements, which proves that using bulk TSSD/TSSP as limits for modelling microscale hydride formation is basically physically inappropriate.

Because hydride precipitation is a phase transformation event, it is sensible to model phase transformation represented by the increase of local misfit lattice strain between parent grain and hydride. To recognise and account for scale-dependent (both bulk and local) hydrogen concentrations measured across different length scales and to smooth the misfit strain transition, three piecewise stages are proposed below during hydride precipitation/dissolution,

7 $${\text{phase}} = \left\{ {\begin{array}{*{20}l} \alpha \hfill & {c < C_{{{\text{bulk}}}} (T)} \hfill \\ {\alpha \,{\text{incorporates}}\,\varepsilon^{{{\text{VM}}}} } \hfill & {C_{{{\text{bulk}}}} (T) < c < C_{{{\text{local}}}} } \hfill \\ \delta \hfill & {c > C_{{{\text{local}}}} } \hfill \\ \end{array} } \right.$$

where *C*_bulk_(*T*) is the TSSP as a function of temperatures between 20 and 300 wppm and 20 to 350 ℃, which is the temperature range considered in this study. *C*_local_ is set to be 3000 wppm which is consistent with local maximum hydrogen concentration measurements for dense microscale hydrides from APT and neutron diffraction [55, 57, 58]. The misfit volumetric strain ***ε***^VM^ is applied differently during precipitation and dissolution,

8 $${\varvec{\varepsilon}}^{{{\text{VM}}}} = \user2{ }\left\{ {\begin{array}{*{20}c} {\begin{array}{*{20}c} { f{\varvec{\varepsilon}}_{{{\text{tot}}}}^{{{\text{VM}}}} {, }} & {{\text{Precipitation}}} \\ \end{array} } \\ { \begin{array}{*{20}c} { - f{\varvec{\varepsilon}}_{{{\text{tot}}}}^{{{\text{VM}}}} ,} & {{\text{Dissolution}}} \\ \end{array} } \\ \end{array} } \right.;\quad f = \frac{{c - C_{{{\text{bulk}}}} \left( T \right)}}{{C_{{{\text{local}}}} - C_{{{\text{bulk}}}} \left( T \right)}}$$

where $f$ is the concentration coefficient to smooth the application of volumetric misfit strain and to reach better convergence during local material properties changing from α to δ and vice versa. The concentration-based misfit strain ${\varvec{\varepsilon}}^{{{\text{VM}}}}$ is applied on zirconium parent grain or hydrides during $\text{precipitation}$ or dissolution, aiming to increase or release the parent-hydride lattice misfit, whereas full misfit strain ${\varvec{\varepsilon}}_{0}^{{{\text{VM}}}}$ is applied to the hydride only for complete precipitation when hydrogen concentration *c* is higher than *C*_local_. This means that the phases in CPFE region are controlled by the local hydrogen concentration. It is noted that the overall hydrogen content in the hydride-matrix system does not change during phase transformation using the scaling method proposed from former work [33].

As a summary, the hydride nucleation is determined in Eq. (7) based on the hydrogen concentration which is a function of the local stress and strain state. The modelling of hydrogen concentration has been shown in former publication [33]. When current concentration reaches the *C*_bulk_, the volumetric misfit strain is introduced gradually according to Eq. (8). And when the concentration reaches *C*_local_, the hydride nucleation event completes and a full hydride forms at this local site/element. It is noted that the first variant from the most common OR1 $\left\{ {0001} \right\}_{{\upalpha }} \left| {\left| {\left\{ {111} \right\}_{{\updelta }} \left\langle {11\overline{2}0} \right\rangle_{{\upalpha }} } \right|} \right|\left\langle {110} \right\rangle_{{\updelta }}$ is used in the hydride precipitation modelling. The variant selection barely affects the hydride morphology in the current length scale of hydride regarding time and location, because the model focuses on the hydride packet modelling dominated by grain interactions, anisotropic thermal expansion and grain-level hydrogen concentrations.

### Tri-crystal and polycrystal model set-up

**Figure 4:**
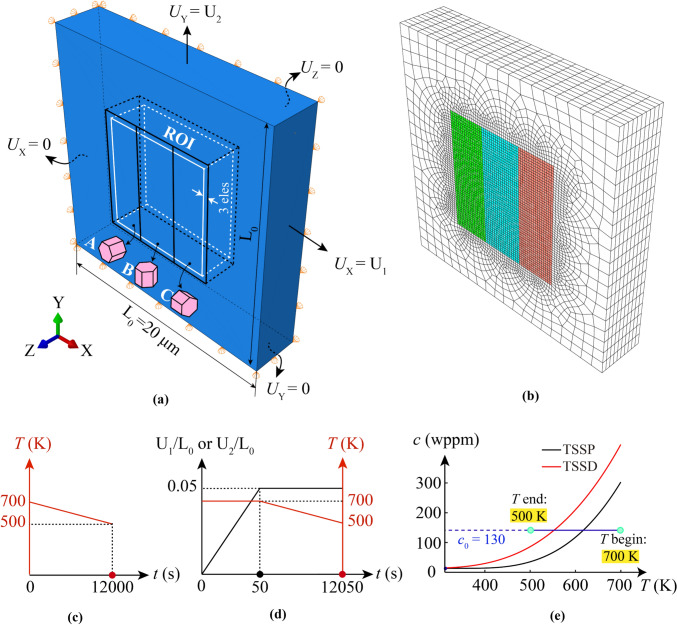
Try-crystal set-up for hydride precipitation. **a** Tri-crystal crystallographic orientations and boundary conditions; **b** FE mesh; **c** spatially uniform cooling condition; **d** thermo-mechanical loads; **e** bulk hydrogen terminal solubility with model set-up.

Tri-crystal and polycrystalline models are introduced in this section. Tri-crystal models are intended to assess the hydride evolution at specific types of grain boundary under uniaxial loading conditions. In Fig. 4a, the tri-crystal model contains three grains with selected (different) crystallographic orientations, i.e. *c* axes along *X*, *Y* and *Z*, respectively. Figure 4b shows the mesh sizes of 0.15 µm and average ~ 1 µm that were applied to the three-grain region and neighbouring region, respectively. The ROI investigated is a subregion of the 3-grain crystal configuration, which is three elements depth away from the boundary of neighbouring region, aiming to avoid the boundary effect from the neighbouring region. The neighbouring region has an isotropic elasto-plastic property with Young’s modulus of 99.3 GPa. The effective inner three grains have anisotropic elasto-plastic properties with the same effective Young’s modulus of 99.3 GPa. Ten elements are assigned along the 3 µm thickness to assess the *Z*-direction hydride precipitation. Thermal cooling in Fig. 4c and thermo-mechanical loading conditions in Fig. 4d are applied, respectively, in the tri-crystal model. Thermal cooling is applied to represent the industrially relevant cooling rate and to investigate the hydride precipitation process. Thermo-mechanical load is separated into two stages: (i) mechanical displacement load inducing plastic deformation with constant temperature; (ii) thermal cooling causing hydride precipitation with constant mechanical load, aiming to investigate the hydride precipitation process when plastic deformation exists. *U*_X_ and *U*_Y_ displacement loads are applied on the tri-crystal model in two separate cases with an overall engineering strain of 5%. The engineering strain is defined as the uniform displacement on either the right or top surface of the whole Tri-crystal model, divided by the original length of *L*_0_ = 20 μm. The resulted effective stress level is constrained by the yielding stress, which is about 350 MPa at room temperature, but changes with temperature, as the elasto-plastic properties of neighbouring region and the inner grains change with the temperature. According to the bulk terminal solubility curves in Fig. 4e, with initial hydrogen concentration *c*_0_ = 130 wppm, cooling from 700 to 500 K leads to hydride precipitation starting at ~ 620 K.

**Figure 5:**
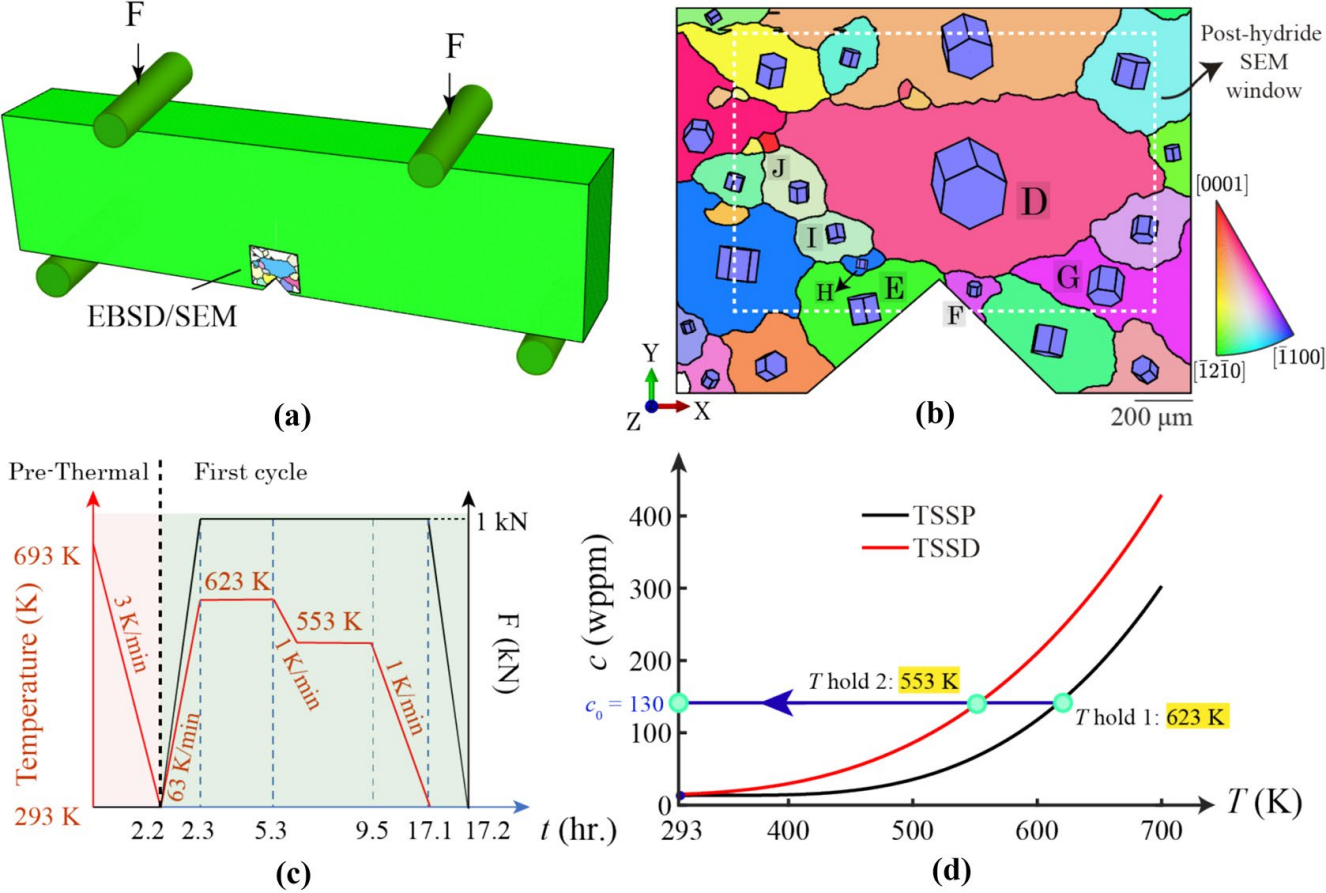
Polycrystalline model set-up. **a** Four-point bending test with ROI scanned by SEM/EBSD; **b** polycrystal with IPFZ colour; **c** thermo-mechanical loading; **d** bulk hydrogen terminal solubility with model set-up.

Figure 5a shows the polycrystalline ROI within the notched sample under four-point bend testing. A notch is included to assess the hydride evolution under triaxial stress conditions with local stress raisers. The polycrystalline case is investigated faithfully with grain crystallography and morphologies from prior SEM/EBSD scanning shown in Fig. 5b. Software package MTEX-5.1.1 in MATLAB was utilised to process the crystallography, grain morphology and GND density *ρ*_GND_ from experimental EBSD scans [59, 60]. The model mesh is shown in Appendix C. Sample preparation and the experimental set-up were introduced in a former study [33]. The sample is subject to industrially relevant conditions and the model reflects true length and time scales, consistent with experiment. In Fig. 5c, the thermo-mechanical load is divided into two parts: (i) pre-thermal cooling; (ii) thermo-mechanical load with a long (~ 15 h) stress-holding period and two shorter (~ 3 h) temperature holding periods, followed by cooling at 1 K/min. Initial hydrogen concentration is 130 wppm. Regarding bulk terminal solubility curves in Fig. 5d, the hydride starts to nucleate at ~ 620 K, but microstructural, heterogeneous, stress distribution will change the local hydrogen concentration due to local stress raisers and grain-to-grain interactions, leading to different hydride precipitation results, which will be shown in Sect. "Notched polycrystal". The triaxial stress/strain state is expected near the crack tip with higher hydrostatic stresses at the vicinity of crack tip. The triaxial stress/strain state is expected near the crack tip with higher hydrostatic stresses at the vicinity of crack tip. The full stress or strain state was shown near the crack tip without hydrides formation, and the microstructure has been proven to be important as the hydrostatic stresses are actually higher at the grain boundary near the notch tip as opposed to the notch tip itself [33].

## Results

Hydride precipitation undergoes complex routes in the polycrystalline configuration, which is affected by the combined effect of locally differing crystallography, anisotropic heterogeneous elasto-plastic behaviour in the hydride and matrix, anisotropic thermal expansion, and external thermo-mechanical conditions. In the following, the tri-crystal configuration is considered first to explicitly show the effect of crystallography and loading conditions on hydride evolution. These insightful analyses are followed by the polycrystalline modelling, which aims to understand the experimentally observed hydride distribution after thermo-mechanical loading.

### Tri-crystal

**Figure 6:**
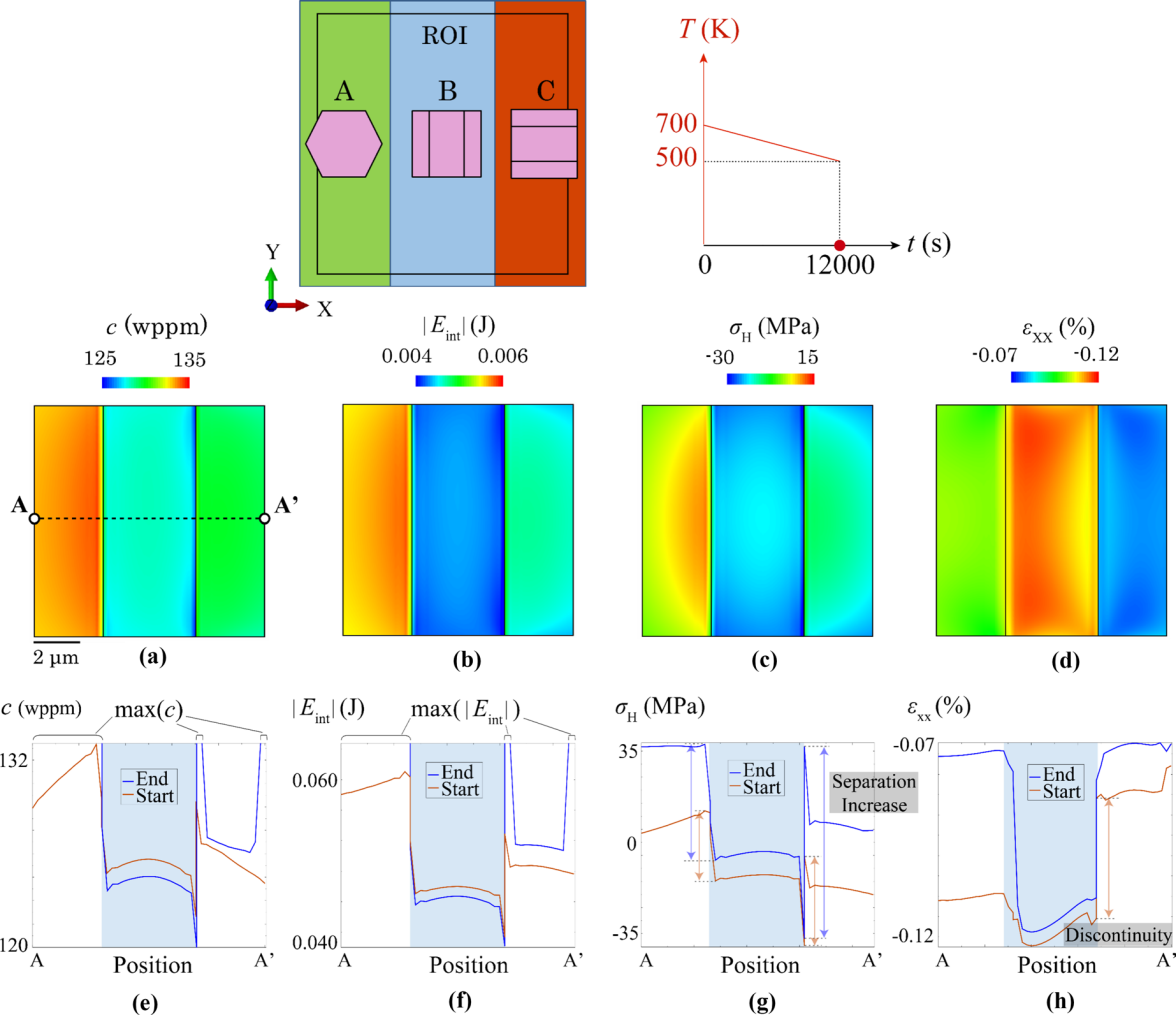
Contour plots of **a** hydrogen concentration c, **b** interaction energy *E*_int_, **c** hydrostatic stress σ_H_, **d** strain component, *ε*_xx_ on ROI surface of tri-crystal configuration and their line profiles along path A-A’ in **e**–**h** at the start and end of hydriding. Shaded area in **e**–**h** highlights the grain boundaries.

In Fig. 6a–d, local contour plots, including hydrogen concentration, *c*, absolute interaction energy, |*E*_int_|, hydrostatic stress, *σ*_H_, and strain component, *ε*_xx_ are shown on *XOY* surface of the ROI in the tri-crystal at the start of hydride precipitation at ~ 620 K. The heterogeneous distribution originates from the anisotropic thermal expansivity of the HCP crystal where *a*-axis thermal expansivity is ~ 2 times that along the *c*-axis. Hydrogen content *c* is high in grain A, relatively low in grain B and medium in grain C. This trend is consistent with the distribution of absolute interaction energy |*E*_int_|. The high interaction energy in grain A results from the high hydrostatic stress that originates from the larger in-plane thermal expansion inside (0001) plane of the HCP crystal, i.e. *a*-axis thermal expansion. Comparing grain B and C, the hydrostatic stress is higher in grain C. This is linked to the dominant thermal expansion direction of B along *X* and C along *Y*, as well as a constrained grain deformation along *X* in grain B. These factors cause a higher in-plane thermal expansion in C, and thus, it has a higher hydrostatic stress than the other two grains. Since the thermal expansivity is isotropic for every direction within the (0001) plane, the thermal strain is distributed similarly into *X* and *Y* components in grain A, leading to its relatively lower strain component *ε*_xx_ along *X*. The highest thermal strain component, *ε*_xx_, is in grain B, whereas relatively lower in C, caused by the high thermal expansivity plane, i.e. (0001) plane, parallel to *X* and *Y* axes, respectively. It is noted that the temperature drop from 700 to 500 K is not large enough to generate plastic strain in the parent grain, which omits the effect of nanoscale defects, such as second-phase particles and interstitial atoms.

Figure 6e–h shows the line profile along path A-A’ for *c*, |*E*_int_|, *σ*_H_, and *ε*_xx_ at the beginning and end of hydride precipitation. The line profiles show the heterogeneous distribution of these quantities, linking to the grain orientations. A sudden drop and rise of hydrogen concentration, *c*, exists at the A-B and B-C grain boundaries, respectively. This correlates well with the trend of |*E*_int_|. A significant drop of interaction energy in grain B is attributed to its compressive hydrostatic stress. According to Eq. (7), compressive hydrostatic stress leads to a negative *E*_C_ which means that the neighbouring cell is pulling out hydrogen atoms from the α-Zr lattice. This leads to a significant decrease of |*E*_int_| and thus low hydrogen content in grain B. The compressive hydrostatic stress in B is caused by its thermal expansive deformation constrained from both *X* and *Y* directions. In grain B, deformation along *X* is limited by A-B and B-C grain interactions and *Y*-axis deformation is constrained by low *c*-axis thermal expansivity. Locally at A-B grain boundaries, the hydrostatic stress changes from tensile to compressive. And the sudden drop of hydrostatic stress near grain B-C boundary in B is caused by the local constraint along *X* from grain C where the *c*-axis in C is parallel to *X* direction with low thermal expansivity. Smoother decrease of *ε*_xx_ happens near A-B grain boundaries, as shown in Fig. 6h, whereas abrupt increase of *ε*_xx_ occurs near B-C.

Compared with the initial state, the hydrogen distribution changes drastically after hydriding, where hydrogen concentrations in A and locally in C reach the maximum value of *C*_local_ = 3000 wppm, which means that these areas on the *XOY* surface reach the hydrogen content limit of hydrides, thus causing the hydride precipitation. Similarly, the absolute interaction energy |*E*_int_| reaches the maximum level under current temperature after hydriding. On the contrary, the hydrogen content in B decreases after hydriding which suggests a hydrogen content transferring from B to A and B to C. The hydride precipitation changes the relatively weak grain-to-grain to stronger phase-grain interaction due to the differences in elastic modulus and slip strength between hydride and matrix (Appendix A), leading to the higher separation of *σ*_H_ and *ε*_xx_ near the A-B and B-C boundaries.

**Figure 7:**
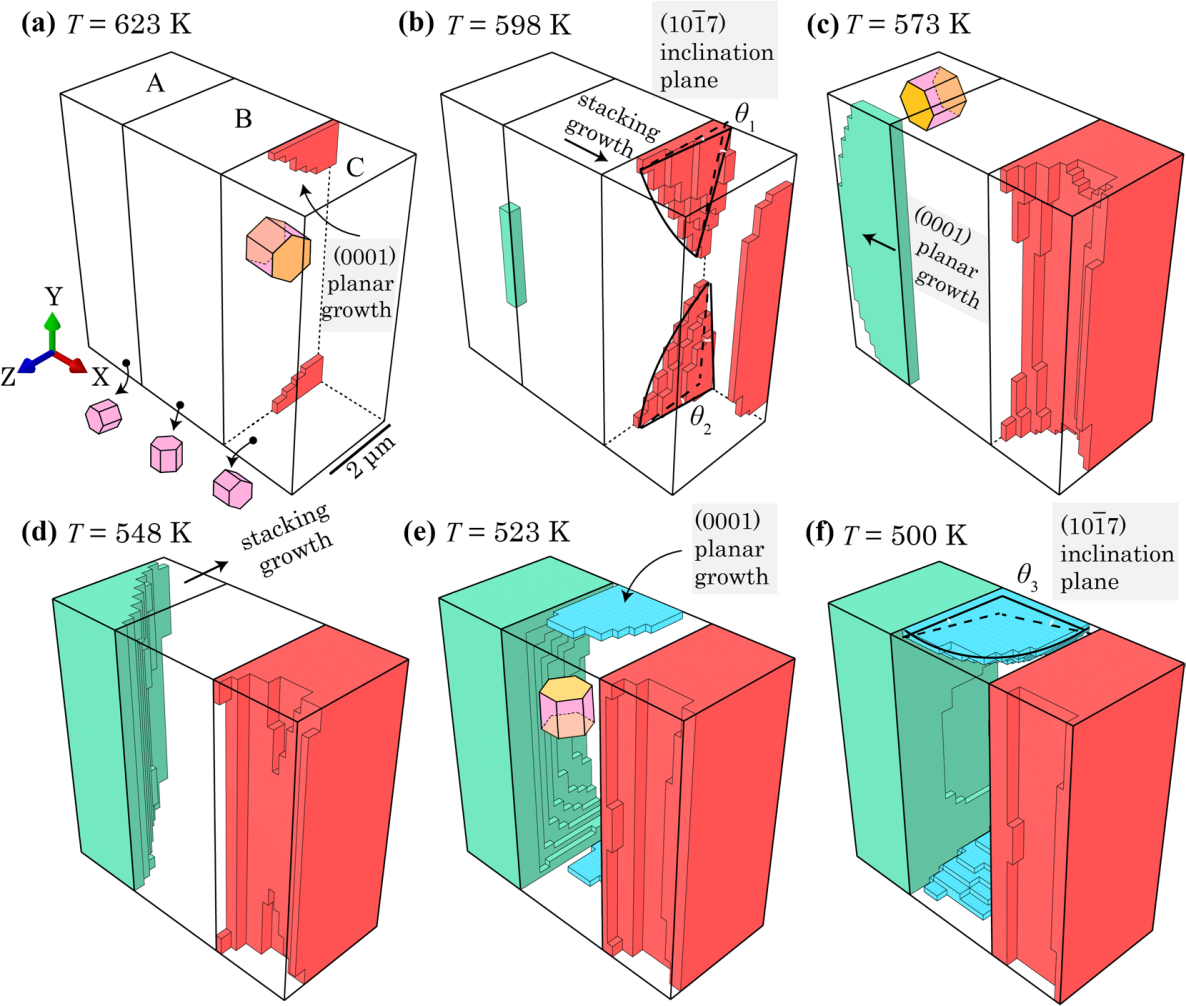
Hydride precipitation process at *T* = **a** 623 K, **b** 598 K, **c** 573 K, **d** 548 K, **e** 523 K and **f** 500 K. Different colours refer to the hydride precipitated inside different grains.

Figure 7 shows the precipitated hydrides at different temperature steps. Two types of hydride growth are observed including planar growth along (0001) plane and the stacking growth perpendicular to the (0001) plane. During the cooling process, two hydride nucleation sites firstly appear near to the B-C grain boundary and at the back of grain C in Fig. 7a. Then the hydride starts to grow inside grain C and another nucleation site occurs at the A-B grain boundary in Fig. 7b. From *T* = 573 K to *T* = 548 K, planar hydride nucleation sites grow and impinge on each other in grain C, whereas a single hydride continued to grow in grain A in Fig. 7c, d, which nearly fills the grains A and grain C subsequently. In the meantime, the hydride in grain B starts to nucleate at the top and bottom plane in Fig. 7e. And planar hydride growth continues in grain B until the end of the thermal load at *T* = 500 K in Fig. 7f. It is interesting to observe that hydride precipitation starts with forming planar morphologies within all three grains in Fig. 7a, c, e and these hydride planes are along the (0001) planes of matrix grains, which is consistent with former observation of nanoscale hydride platelets following the basal plane of the matrix in HCP α-Zr [10, 20]. This results from the volumetric misfit strain with the higher *c*-axis component, 1.6 times the component along *a*-axis [16], which triggers the high compressive stress within the basal plane when hydrides form, therefore constraining the hydrides to grow along the basal plane. Besides, consistent with a previous study [46], the planar shape hydride leads to the higher hydrogen content at the hydride front than the planar side, leading to more preferential growth inside the basal plane, i.e. along the *a*-axis.

It is noted that the α/δ interfacial strain is likely to be important at the atomic length scale, but we argue this is a second-order effect at the grain scale. The precipitated hydride interacts with the parent and neighbouring grains, which leads to the redistribution of local misfit strain, subsequent redistribution of hydrogen and formation of hydride clusters. As demonstrated by nano-beam diffraction experiments [61], the measured interfacial strain is lower than the hydride precipitation volumetric strain from Carpenter (1973). This means that the hydride-matrix interfacial strains are dominated by the volumetric misfit strain (or phase transformation strain) and subsequent stress re-equilibration. In the current model, results indicate stacking growth of planar hydrides during hydride precipitation shown in Fig. 7b, d, f for the hydrides in all three grains. In Fig. 7b and f, respectively, growth front of the hydride stacking in grain B and C starts to be steeper, showing a shallower gradient that has an angle of deviation, i.e. *θ*_1_, *θ*_2_, and* θ*_3_, from the basal plane, ranging from 10° to 20°, which is consistent with the observed 14.7° angle between (0001) plane and habit plane of (10$\overline{1 }$7) in hydride packets. This results from the α-δ interaction as well as the uneven distribution of hydrogen around platelet-type hydrides. It is noted that the habit plane formation is a temporary state and the hydride morphology changes in (c)–(f). Essentially, the tri-crystal case is a contrived example aiming to study comparison of hydride precipitation between thermal and thermo-mechanical load. In the current tri-crystal case, when the growing of hydride packets is close to the boundary condition (z-axis fixed), the interaction between the precipitated hydride packet and constrained boundary condition becomes severe; afterwards, the stacking hydride forms following the hydrostatic stress localisation caused by the constraints from the boundary conditions outside of the ROI. The z-axis constraint at back of the model also acts as a stress-raiser contributing to the hydrostatic stress localisation and hydride nucleation.

It is also noted that {10$\overline{1 }$7} morphological direction was observed in both intragranular and intergranular cases. The intragranular hydride is usually initiated by the sub-grain defects, such as second-phase particles and interstitial defects, which is due to be studied in further works.

Overall, under thermal cooling conditions without external load, the hydride growth shows a strong relationship with local crystallography due to the anisotropic thermal expansivity and the anisotropic misfit strain during hydride formation.

**Figure 8:**
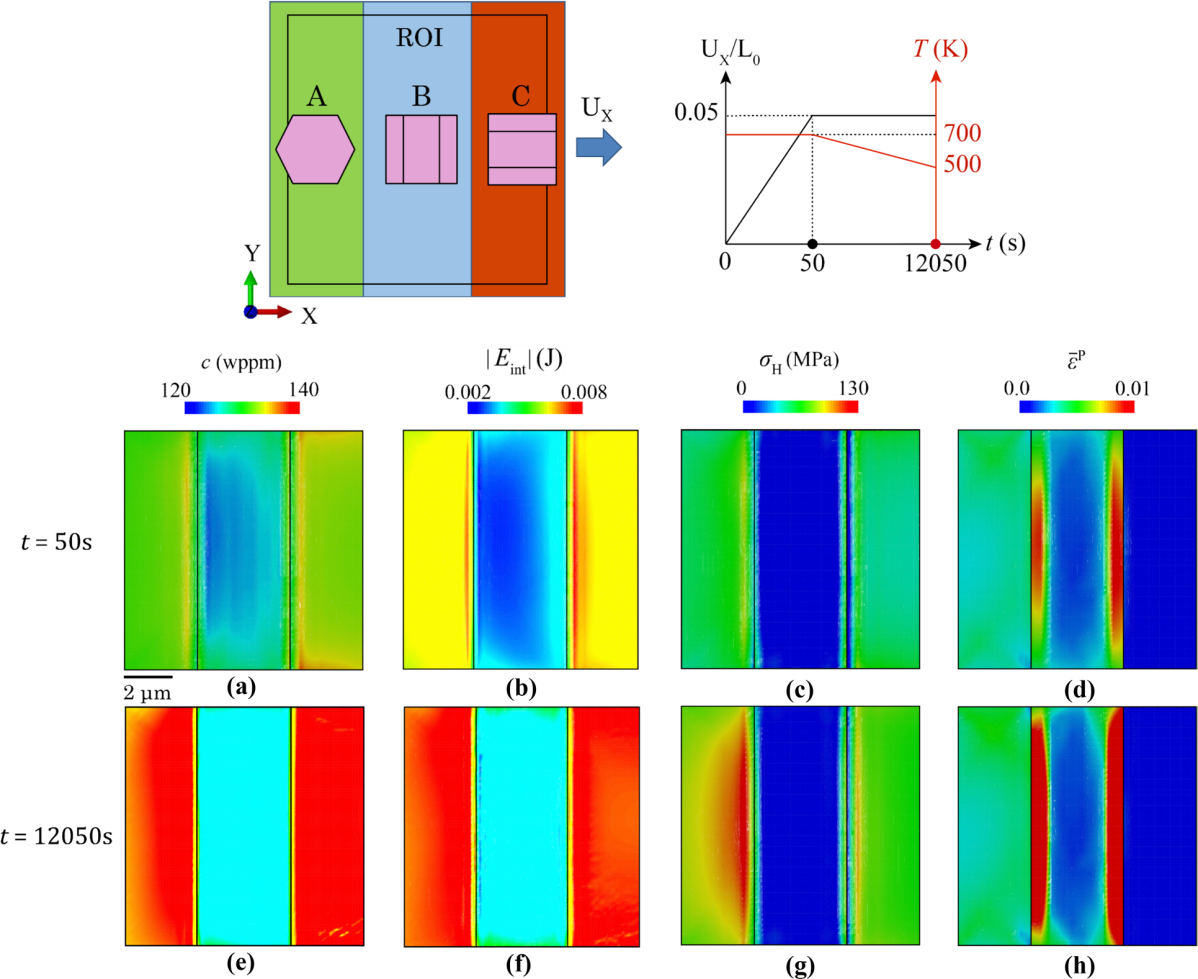
Contour plots of **a** and **e** hydrogen concentration c, **b** and **f** interaction energy *E*_int_, **c** and **g** hydrostatic stress *σ*_H_, **d** and **h** accumulated plastic strain ${\overline{\upvarepsilon } }^{\text{p}}$ at the end of mechanical (**a**–**d**) and thermal step (**e**–**h**).

In the combined thermo-mechanical loading, the external load was introduced into the tri-crystal in the first stage to investigate its effect on hydride growth under isothermal conditions. At the end of the load-up stage in Fig. 8a, high hydrogen contents *c* are shown near both A-B and B-C grain boundaries, consistent with the distribution of absolute interaction energy |*E*_int_| in Fig. 8b. This results from the high hydrostatic stress at the two grain boundaries in Fig. 8c. Gap regions of lower hydrostatic stresses are observed between the concentration areas and the nearby grain boundaries. This is linked to the local high plasticity ${\overline{\varepsilon }}^{\text{p}}$ near grain boundaries in grain B shown in Fig. 8d. At these plastic accumulation regions, incompressibility dominates for metallic HCP materials and lattice triaxial stretching (hydrostatic strain) is small [62], which leads to lower or even compressive hydrostatic stress near the two grain boundaries, therefore low |*E*_int_| and *c* in grain B. Note that a purely elastic analysis leads to a concentrated hydrostatic stress at the stress raisers [63].

At the end of the thermal step, high hydrogen content >  ~ 140 wppm occurs in grains A and C in Fig. 8e, and the contour plot is consistent with |*E*_int_| in Fig. 8f. Relatively higher hydrostatic stress was observed in grains A and C in Fig. 8g driven by the plasticity accumulation near grain boundaries in B. Relatively low plastic deformation occurred in grain A and no plasticity in grain C.

**Figure 9:**
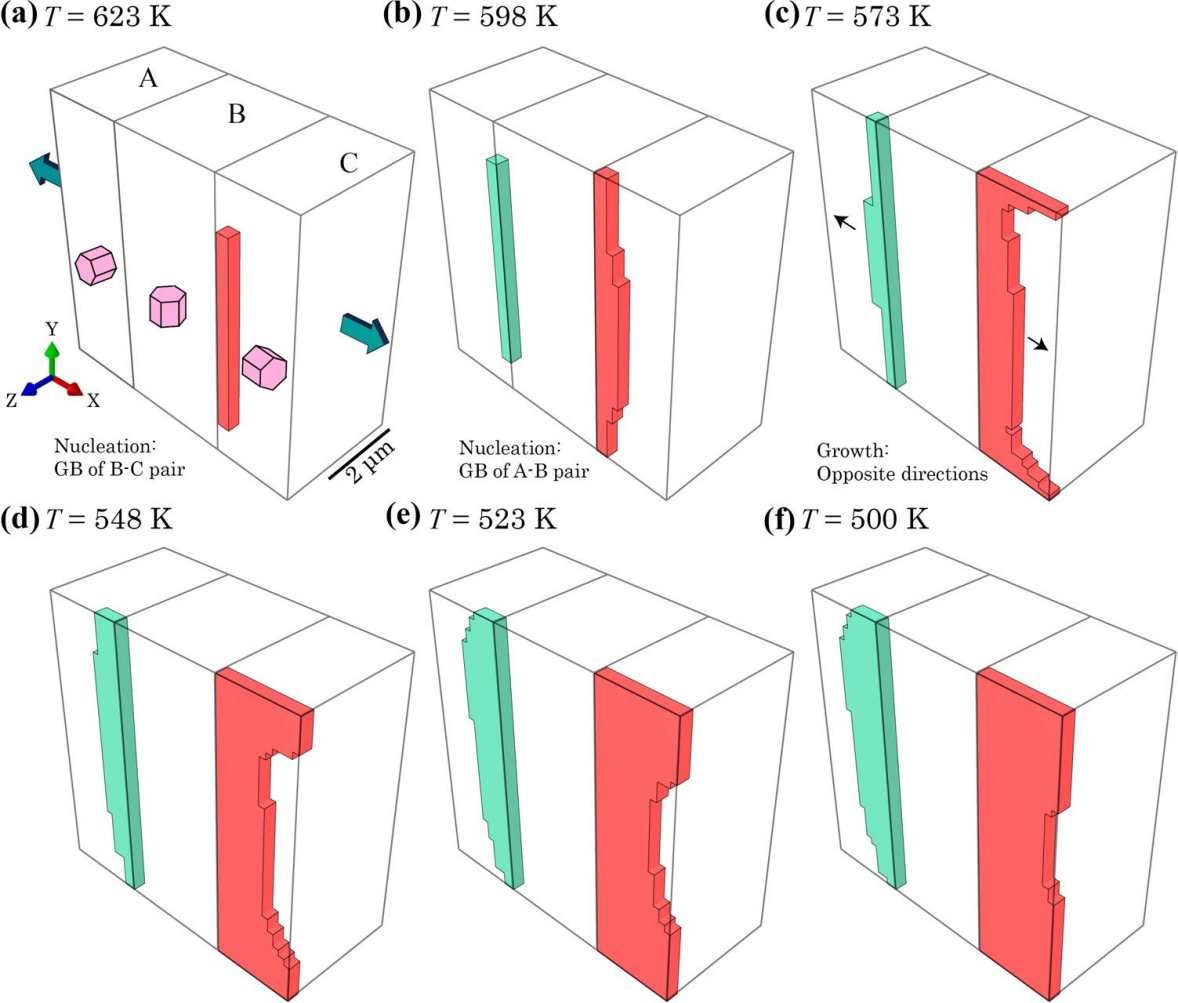
Hydride evolution during thermal cooling process at *T* = **a** 623 K, **b** 598 K, **c** 573 K, **d** 548 K, **e** 523 K and **f** 500 K after displacement step along X.

Hydride growth is explicitly shown in Fig. 9. The hydride first nucleates in grain C at the B-C boundary in Fig. 9a, followed by the nucleation in grain A at the A-B boundary in Fig. 9b. The nucleation sites are consistent with the high hydrogen concentration on *XOY* surface in Fig. 8. The growth of two hydrides in opposite directions in Fig. 9c is caused by the low hydrostatic stress and high (incompressible) plastic deformation near the two grain boundaries in grain B. The continuing planar hydride growths are observed in Fig. 9e–f, where the free surface condition leads to higher in-plane stresses, thus higher |*E*_int_| at the hydride front in the plane, which favours the hydride growth along the planar direction in grain A and C. At the end of hydride growth in Fig. 9f, the hydride reaches the top and bottom of grain A, which is consistent with the lower hydrogen content in Fig. 8e. It should be noted that when the local hydrogen content reaches the bulk hydrogen limit, *C*_bulk_, it does not ensure the hydride precipitation, but the local high hydrogen *c* > *C*_local_ (3000 wppm) needs to be reached during the increase of volumetric misfit strain, ***ε***^VM^ in Eq. (6).

**Figure 10:**
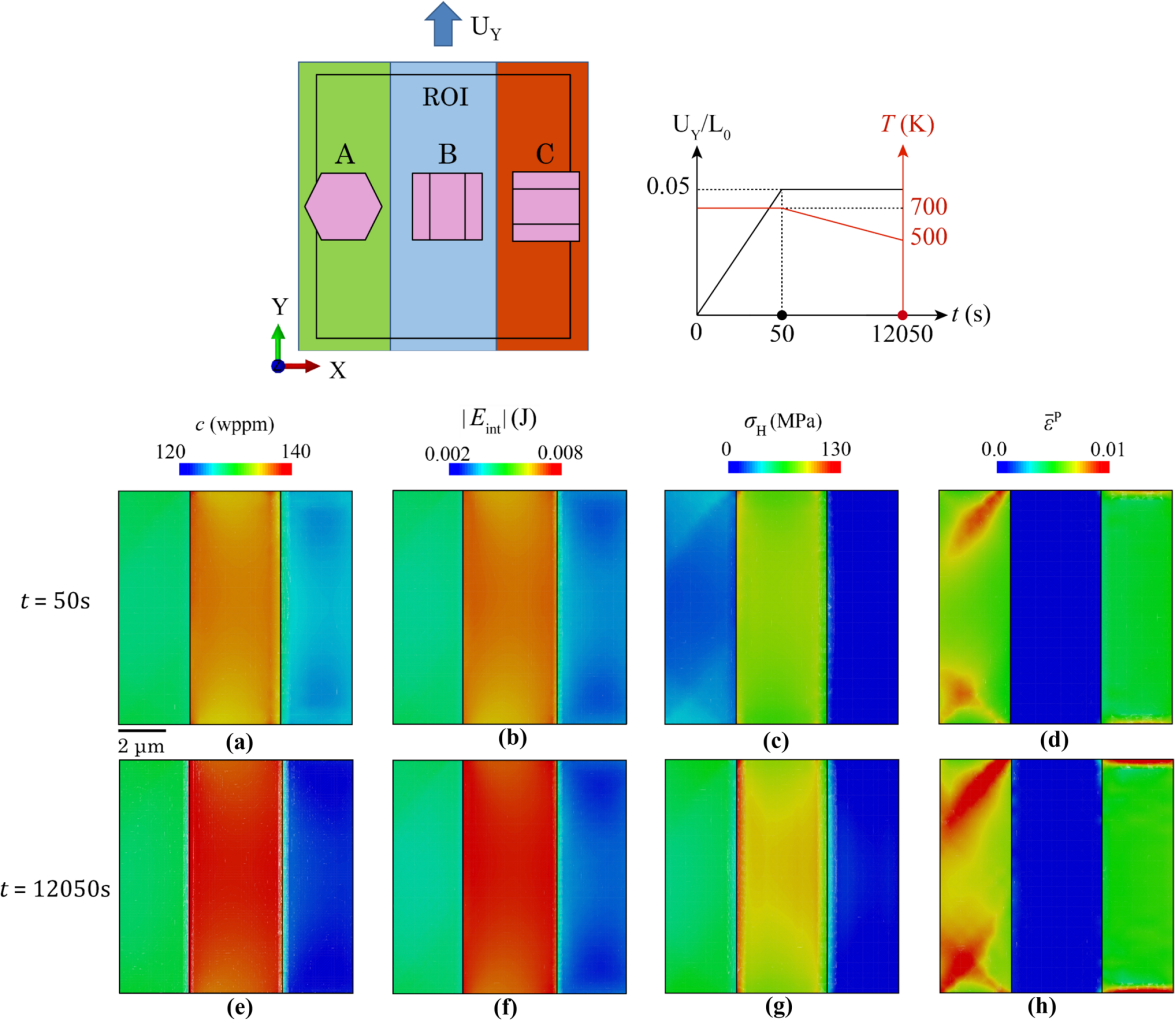
Contour plot of **a** and **e** hydrogen concentration c, **b** and **f** interaction energy *E*_int_, **c** and **g** hydrostatic stress *σ*_H_, **d** and **h** equivalent plastic strain ${\overline{\upvarepsilon } }^{\text{p}}$ at end of Y-displacement (**a**–**d**) and thermal step (**e**–**h**).

In a separate analysis with initially mechanical loading along *Y*, the contour plots change completely due to differing elasto-plastic deformation for the different crystal orientations and slip system activations. At the end of the load-up stage, the hydrogen localises in grain B and relatively lower hydrogen concentration is observed in grains A and C in Fig. 10a, consistent with the distribution of absolute interaction energy |*E*_int_| in Fig. 10b. The hydrostatic stress shows a similar pattern in Fig. 10c due to the higher *c*-axis elastic modulus in grain C along *Y*. The plastic strain accumulated in grains A and C is shown in Fig. 10d.

At the end of thermal cooling,* c* and |*E*_int_| increase in grain B, whereas they drop in grain C in Fig 9e and f, respectively. This is mainly driven by the higher difference of hydrostatic stress between grain B and C in Fig 9g. Plastic deformation is significantly higher in non-hydride-containing grains A and C, due to their preferential crystal orientation for prismatic slip. Hydride growth in grain C does not follow the basal plane hydride nucleation, which was shown in thermal only case. This is resulted from: (i) the extra strain load along *X*, leading to high stresses and strain from elasticity in ‘hard’ grain C with *c*-axis parallel to loading direction; (ii) free surface condition, leading to in-plane stress condition and thus higher hydrostatic stress on the surface to reach the same amount of strain applied; (iii) hydride precipitation in Fig. 9a and b triggers the strong hydride-matrix interaction and the higher hydrostatic stress and strain at the hydride front, but also constrained on the surface. These combined factors lead to the high interaction energy |*E*_int_| on the surface which drives the constrained hydride growth on the free surface of grain C.

**Figure 11:**
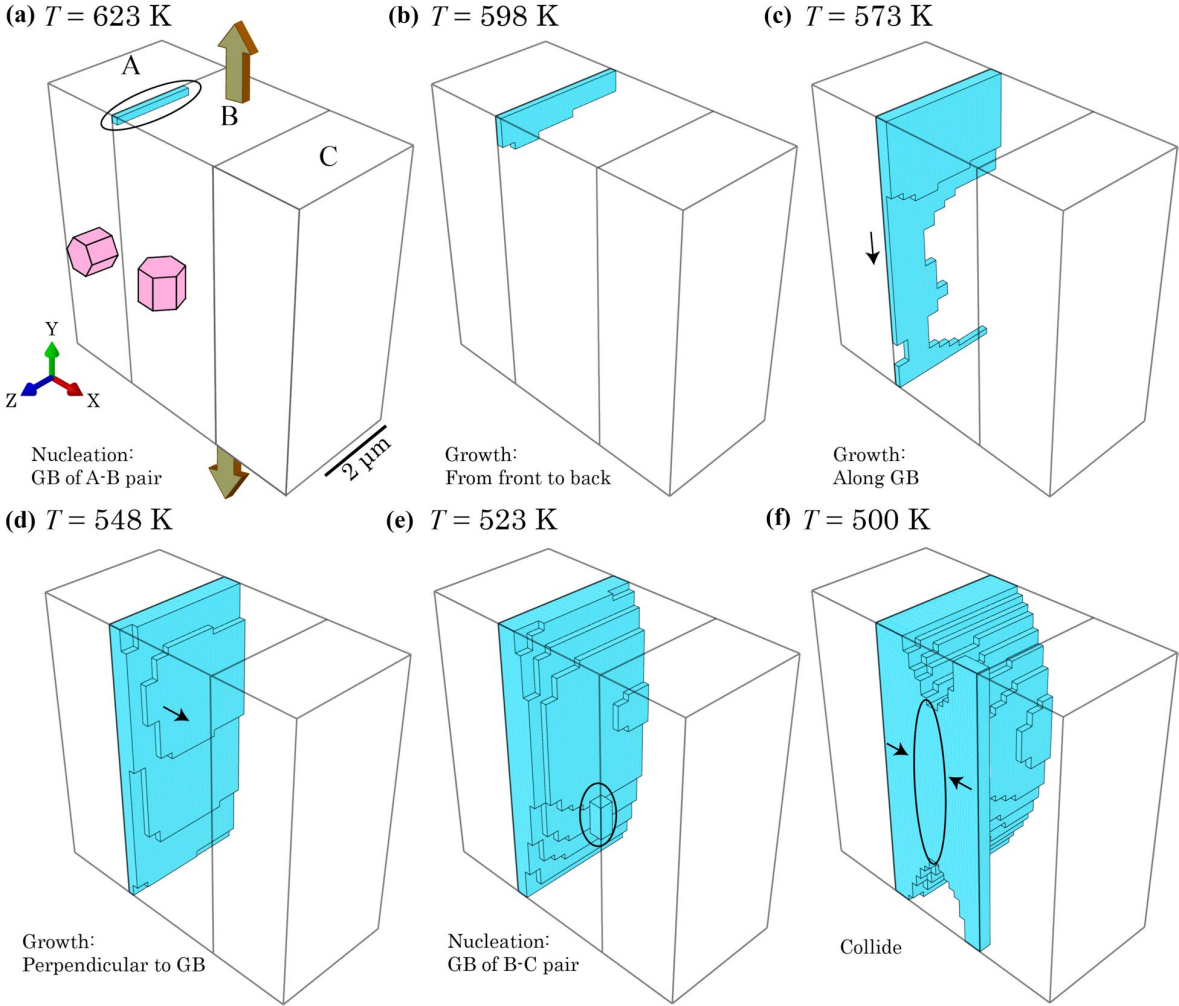
Hydride evolution during thermal cooling process at *T* = **a** 623 K, **b** 598 K, **c** 573 K, **d** 548 K, **e** 523 K and **f** 500 K after displacement step along Y.

Figure 11a shows the hydride nucleates on the topside of the A-B grain boundary in grain B, followed by a planar growth from front to back and from top to bottom in Fig. 11b, c. The planar growth continues from top to bottom with non-uniform distribution of hydrides due to spatial gradient of |*E*_int_| in the out-of-plane direction. Parallel growth of hydride to the grain boundary is constrained by the grain interactions from extra strain load along *Y* where highest strain and hydrostatic stress occurs in Fig. 10c and d, leading to high |*E*_int_| and thus high hydrogen concentration near grain boundaries. The stacking growth of planar hydrides occurs along a direction perpendicular to the grain boundary plane in Fig. 11d, in the meantime planar growth fills the whole A-B grain boundary. Figure 11e shows another nucleation site occurs at the B-C boundary and more stacking growth continues out of the A-B boundary. Finally, a planar hydride forms at the front surface of grain B due to the colliding of two hydrides from different nucleation sites, consistent with the hydrogen concentration distribution in Fig. 10e.

The hydride growth shows completely different patterns for thermal cooling versus thermo-mechanical conditions. Planar hydride growth along basal plane is observed under only thermal cooling, whereas non-basal hydride growth occurred in both thermo-mechanical cases along either *X* or *Y* direction. This originates from the mechanical load at the initial stage, which changes the local stress and strain state due to grain interactions, and heterogeneous dislocation slip with respect to each of the three different grain orientations. These local mechanical states constrained the growth direction of hydride; meanwhile the hydride growth constantly changes the local mechanical state, as well as the interaction energy |*E*_int_| and local hydrogen concentration, near the hydride front.

Furthermore, dominant hydride growths are observed in grain A loaded along *X* and in grain B loaded along *Y*. This is caused by the elasto-plastic anisotropy where *c*-axis direction has a higher elastic modulus than the *a*-axis and a higher resistance to plastic deformation, leading to higher local hydrostatic stresses when loaded along the *c*-axis direction. This means that hard grains with *c*-axis near parallel to the remote load are susceptible to hydride nucleation and growth. On the contrary, the soft grains with *c*-axis perpendicular to the loading direction show much greater plastic deformation. Incompressibility dominates the local region with high plastic deformation, which indicates the stress state is predominantly shear in turn suggesting low hydrostatic stress, leading to low |*E*_int_| and lower hydrogen concentration and hence less hydride nucleation and growth. This means that local plasticity accumulation leads to less potential for hydride nucleation. Another observation is that when a hydride nucleates at the front surface, the hydride starts to grow preferentially along the surface plane. Since *σ*_zz_ = 0 on the surface, to reach the same amount of strain applied, the in-plane stress condition results in higher *σ*_H_, |*E*_int_| and therefore a higher hydrogen content at the free surface. It is also noted that the tri-crystal configuration is a contrived case with specific design of grain crystallography and morphology. Practical polycrystalline microstructures contain differing grain morphologies and strong-textured crystallographic orientations, generating more complex hydride growth [13].

### Notched polycrystal

**Figure 12:**
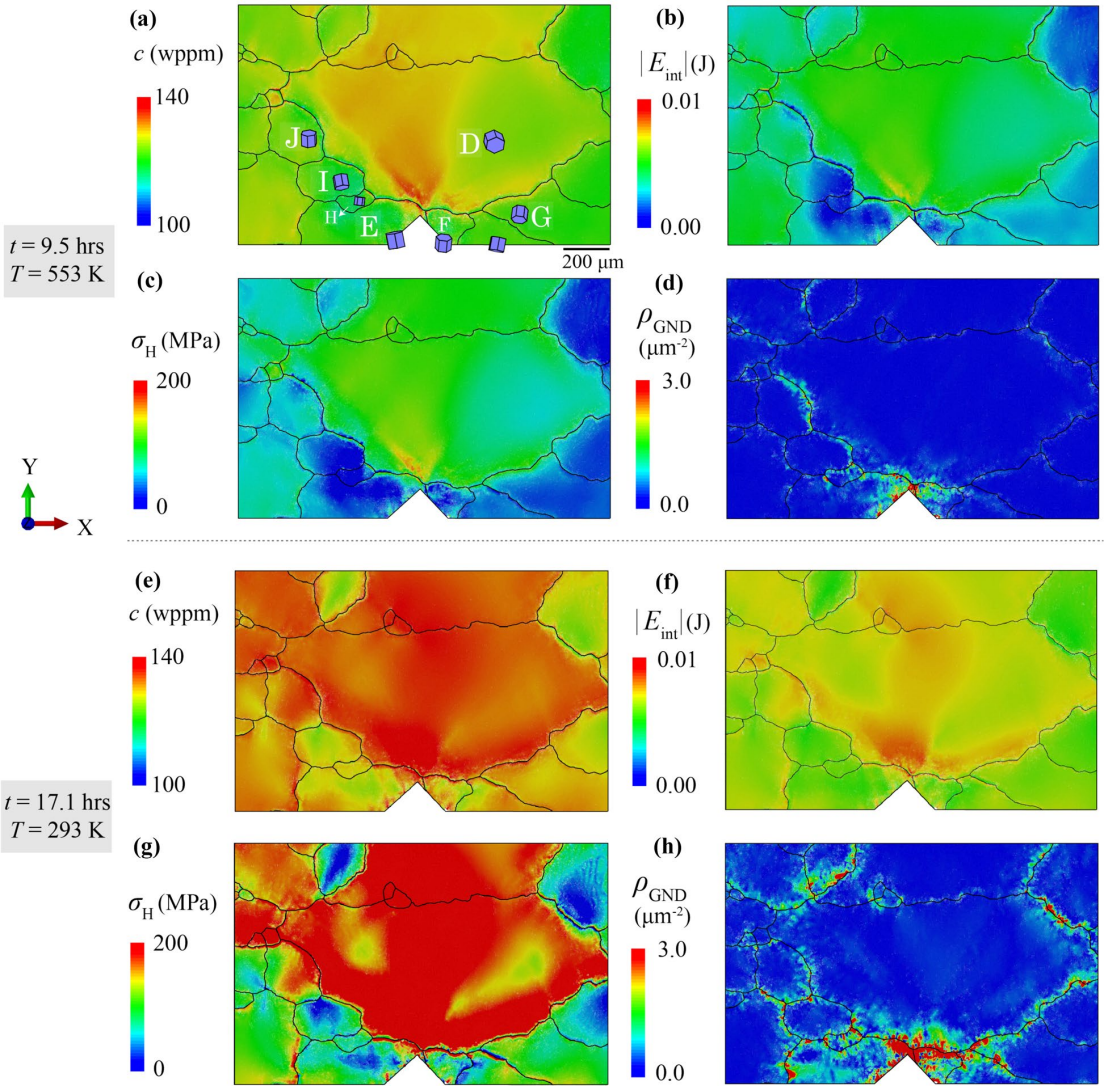
Contour plots of **a** and **e** hydrogen content c, **b** and **f** absolute interaction energy |*E*_int_|, **c** and **g** hydrostatic stress *σ*_H_ and **d** and **h** *ρ*_GND_ at the start of second thermal cooling (*t* = 9.5 h, **a**–**d**) and at the end of temperature cooling (*t* = 17.1 h, **e**–**h**).

In the notched polycrystal case, the ROI is at the notch and the effects of a triaxial stress state and stress gradient on the local hydride growth are studied under industry-relevant thermo-mechanical load with temperature ranging from 293 to 623 K and stress intensity factor *K* ~ 10 MPa/$\sqrt{\text{m}}$ [33]. To understand the predicted hydride precipitation in the polycrystal, contour plots are shown in Fig. 12 for hydrogen content, *c*, absolute interaction energy, |*E*_int_|, hydrostatic stress, *σ*_H_ and GND density, *ρ*_GND_. At the start of the second thermal cooling stage, high hydrogen concentration *c* occurred at the D-E boundary and in D in Fig. 12a, consistent with the distribution pattern of interaction energy |*E*_int_| in Fig. 12b, and hydrostatic stress* σ*_H_ in Fig. 12c. The GND density *ρ*_GND_ in Fig. 12d indicates the high plasticity (gradients) at the notch tip and near the D-E grain boundary, which is the result of both pre-load thermal cooling and plastic deformation.

At the end of thermal cooling, the hydrogen distribution pattern changes dramatically and the hydrogen concentrates near the grain boundary between D and its neighbouring grains, which is different from the former tri-crystal study where patterns barely changed during thermal cooling [33]. |*E*_int_|, *σ*_H_ and *ρ*_GND_ are also localised near to these grain boundaries, whereas *ρ*_GND_ concentrates on the other side of the grain boundaries from |*E*_int_| or *σ*_H_. As explained in the tri-crystal case, when GND density develops on one side, high triaxial tensile strain occurs at the other side of the grain boundaries to compensate or balance the change of the lattice distortion, i.e. curvature change curl(${{\varvec{F}}}^{\text{p}}$) [38]. Locations with high hydrogen content result in precipitated hydrides with higher strength than the matrix, leading to high stress concentrations. The effect of plasticity (low hydrostatic stress) drives the hydrogen away from the notch and inhibits the hydride precipitation at the neighbouring boundaries of D, where the high interaction energy |*E*_int_| occurs.

**Figure 13:**
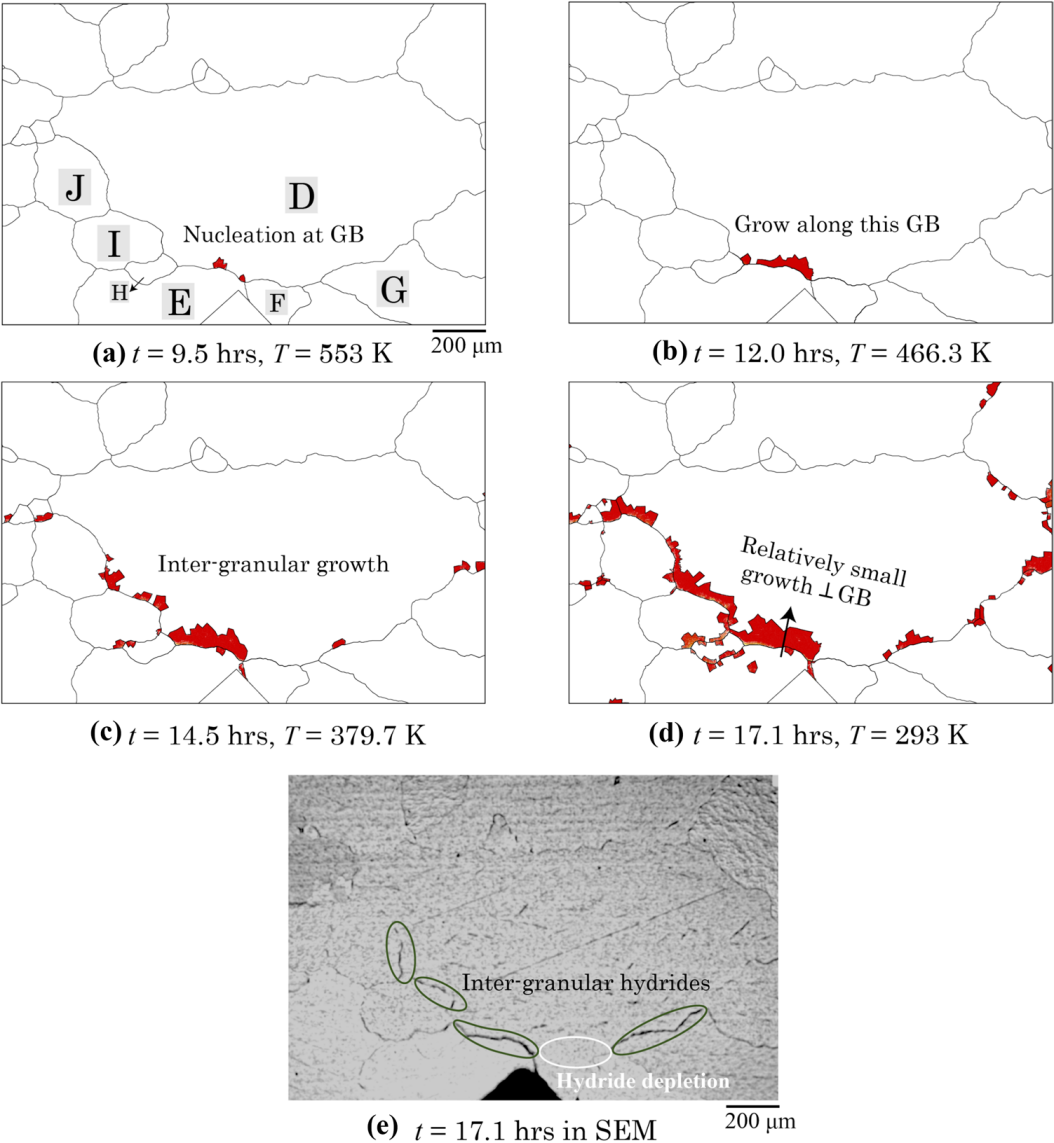
The hydride evolution in polycrystal during the secondary thermal cooling process at *t* = : **a** 9.5 h, **b** 12.0 h, **c** 14.5 h, **d** 17.1 h, comparing to **e** experimental results at *t* = 17.1 h.

Figure 13 shows the explicit predicted hydride evolution in the practical polycrystal system. The hydride nucleates near the D-E grain boundary in Fig. 13a due to the first thermal cooling from 623 to 553 K. The intergranular hydride growth continues in Fig. 13b along the same grain boundary. This results from the plastic deformation occurring at the bottom side of D-E grain boundary, creating low or negative hydrostatic stress where there are high levels of plastic deformation and corresponding high hydrostatic stress on the top side of this grain boundary, shown in the contour plots of Fig. 12c, d, consistent with our former study [33]. This leads to high |*E*_int_| and high hydrogen content near this grain boundary, similar to the tri-crystal results in Fig. 8a–d. This demonstrates that local plasticity and GND increase are directly linked to the intergranular hydride nucleation, which constrained the growth along the grain boundary in Fig. 13b on the grain D side of the boundary.

Figure 13c shows more intergranular hydrides growing along the grain boundaries between D and its neighbouring grains, especially the ones close to the notch. More nucleation sites are observed at the locations where high |*E*_int_|, *σ*_H_ and *ρ*_GND_ were observed in Fig. 12b–d. Intragranular growth of hydrides perpendicular to the D-E grain boundary is observed, where initial hydride nucleation is shown in Fig. 13a. However, the length scale of the intragranular grain growth is relatively smaller than that growing along the grain boundary. This is consistent with the tri-crystal case presented where the hydride front progresses quicker along the grain boundary than the hydride plating a grain boundary and growing perpendicular to the grain boundary. To explicitly show the hydride growth in grain ‘D’, the 3D morphology of the hydride formation near ‘D-F’ boundary is shown in Appendix D to include the thickness direction, and it could be clearly shown that the hydride growth follows the orientation relationship with the basal plane which was observed in tri-crystal case.

Grain boundary hydrides predicted by the model are consistent with those from SEM observations in Fig. 13e, especially in the circled grain boundaries between grain D and its neighbouring grains. It is noticed that a hydride depletion region occurs near the D-F grain boundary, which is predicted by the modelling. This results from the relatively lower or negative hydrostatic stress *σ*_H_ and high *ρ*_GND_ near the D-F grain boundary in Fig. 12c and d compared with the ones near D-E, which drives the hydrogen out of this region leading to the relatively low |*E*_int_| and hydrogen content in Fig. 12 (ambo). It is noted that the hydrogen content near the D-F grain boundary in Fig. 12e exceeds the bulk hydrogen limit *C*_bulk_ but is much less than the *C*_local_, which has not reached the limit for hydride nucleation.

## Discussion

### Effect of twin

In this section, the hydride evolution is investigated within a deformation twin-embedded microstructure, compared with an identical but non-twin microstructure. Stored energy localisation is linked to the twin nucleation site and therefore a twin-embedded polycrystal model is reconstructed. The twin model mesh and identification of twin orientations, which satisfies the twin morphological direction are shown in Appendix E.

**Figure 14:**
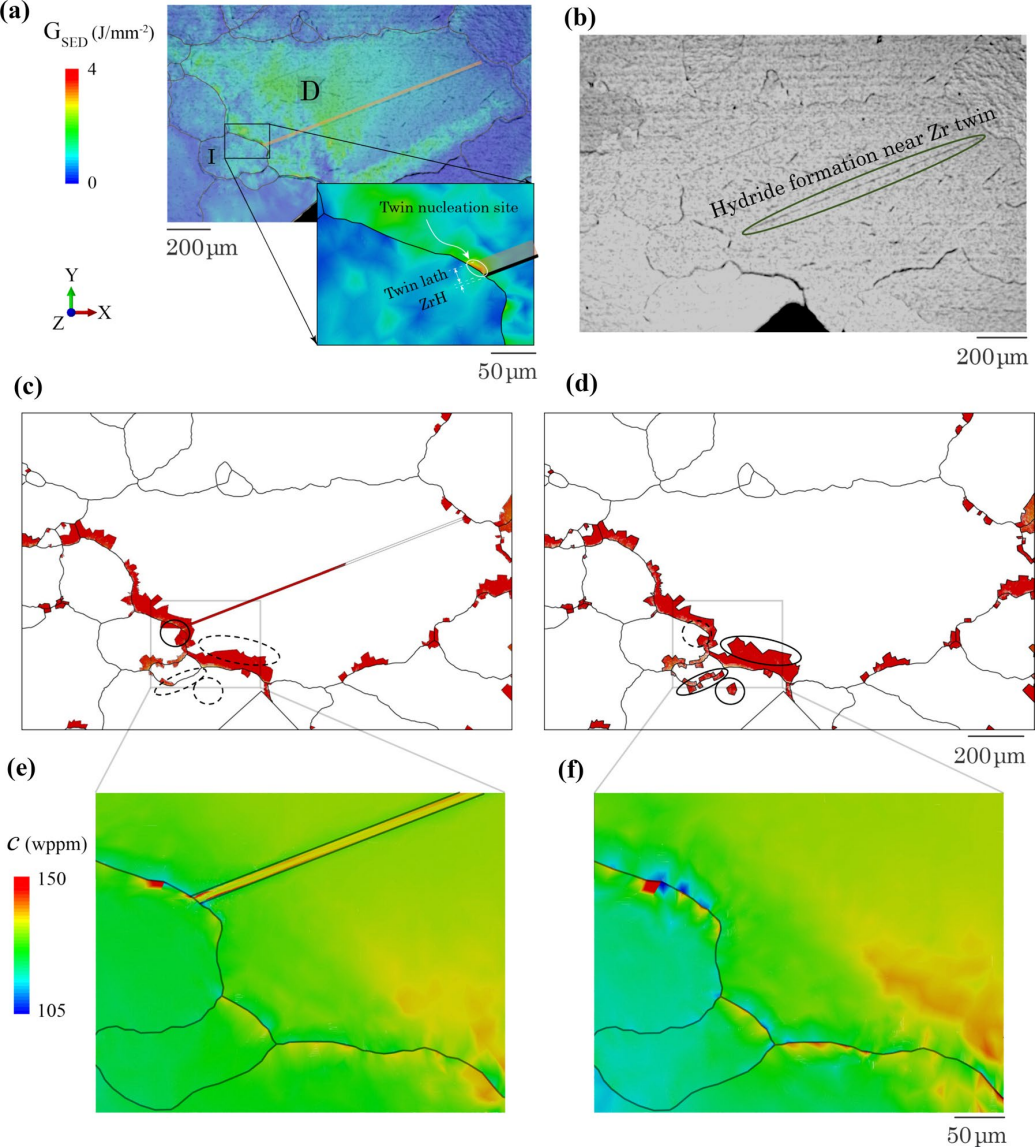
Effect of twin on hydride formation. **a** Twin nucleation overlaying experimental SEM observation; **b** experimental hydride formation at vicinity of twin; predicted hydride evolution **c** with or **d** without twin incorporated at *t* = 17.1 h; zoom-in region showing hydrogen concentration near D-I grain boundary at *t* = 9.5 h **e** with or **f** without twin.

In the material preparation of the polycrystalline sample in Fig. 5b, large blocky-α grains were generated. A former study showed that this procedure led to the formation of thin layer twins within the blocky-α grains [64]. These thin layer twins across the large grains are expected to influence hydride nucleation and growth during thermal cooling. Stored energy density, *G*_SED_, was demonstrated as a good indicator of twin nucleation sites in HCP polycrystals [42]. In Fig. 14a in a non-twin model (at t = 9.5 h), high *G*_SED_ is observed at the D-I grain boundary and is therefore a predicted twin nucleation site. It is interesting that the predicted site is exactly at the crossing point of the hydrided twin and D-I grain boundary in Fig. 14a. This validates the twin nucleation and the twin-hydride interaction where hydrides form at the twin in experiment in Fig. 14b.

Hence, hydride evolution is studied within the reconstructed twin-embedded polycrystal model. The model containing the twin in Fig. 14c shows hydride formation along the twin region at the end of cooling, driving the less local hydride formation in the dot-circle region where the hydride grows in the non-twin model in Fig. 14d. This is consistent with former studies where hydrogen content is relatively higher inside twins [45]. The hydride shrinkage results from the redistribution of hydrogen concentration due to the presence of twin at the beginning of hydride growth at *t* = 9.5 h, results of which are shown in Fig. 14e, f. Localised hydrogen content is observed inside the twinned region with lower hydrogen concentration near the D-I grain boundary in Fig. 14e. On the contrary, much lower hydrogen concentration *c* occurs in grain D without the twin and higher *c* near D-I grain boundary shown in Fig. 14f.

### Residual stress from cooling

In this section, the effect of residual stresses from the thermal cooling process is investigated. A recent study showed that the residual stresses in zirconium crystals were measured in the range of − 200 to 200 MPa with a standard deviation of 52 MPa from 3D synchrotron XRD [65]. In this study, the pre-thermal cooling process is removed from Fig. 5c, which will affect the residual stresses, GND and plasticity accumulation during cooling, therefore influencing the hydride precipitation, which is compared to the case with pre-thermal cooling.

**Figure 15:**
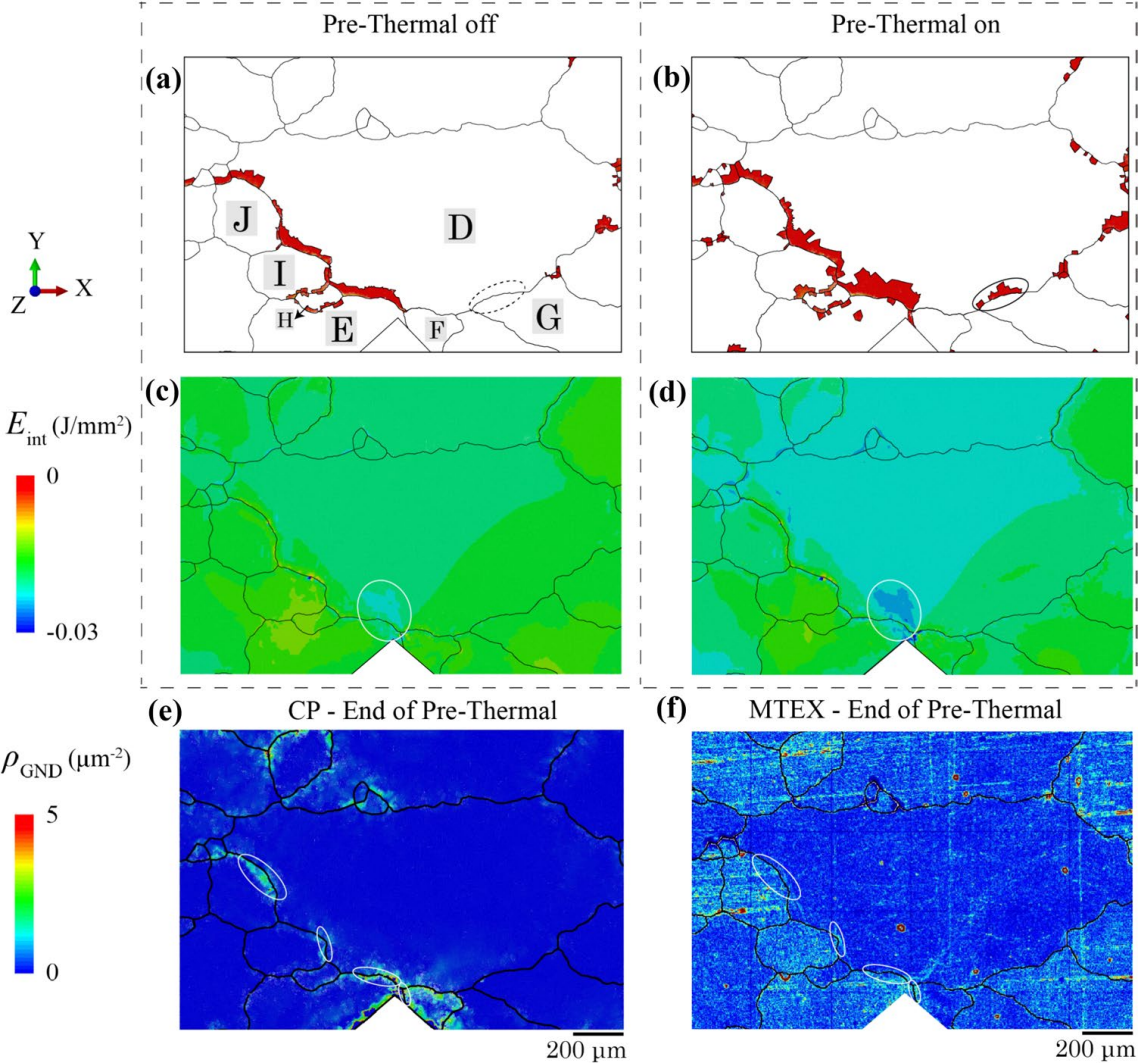
The effect of pre-thermal cooling condition from Fig. 5c, including the **a** and **b** distribution of hydrides at *t* = 17.1 h and **c** and **d** distribution of *E*_int_ at *t* = 9.5 h between **a** and **c** no pre-thermal cooling and **b** and **d** pre-thermal cooling conditions. **e** CP and **f** MTEX results are compared for *ρ*_GND_ at the end of pre-thermal cooling process.

The thermal cooling at the end of sample-preparation process in Fig. 5c for homogenising hydrogen content can have a significant impact in the polycrystalline responses, since the large temperature drop leads to the intrinsically high residual stresses within the microstructure [66]. In the case of no pre-thermal cooling process, the model begins with no hydrides and hydrides are fully dissolved at high temperature, the hydriding only starts in the thermal cooling step. When the pre-thermal cooling process is not included, the growth of intergranular hydrides is weaker without the pre-thermal cooling condition in Fig. 15a, specifically showing an absence of hydrides near the D-G grain boundary, compared with the ones in Fig. 15b considering pre-thermal cooling. No pre-thermal condition leads to relatively higher negative interaction energy *E*_int_ in Fig. 15c, i.e. lower |*E*_int_|, whereas high |*E*_int_| existed near to the D–E grain boundary in Fig. 15d. This reduces the hydride growth as lowering the |*E*_int_|, and corresponding hydrogen concentration leads to less local area satisfying the hydride formation limit *C*_local_.

The *ρ*_GND_ distribution from CP in Fig. 15e shows reasonable agreement with computed MTEX results in Fig. 15f after the pre-cycle stage, especially the* ρ*_GND_ concentration near the D–J, D–I and D–E grain boundaries. The determination of *ρ*_GND_ from the sample free surface leads to similar magnitude and distribution of *ρ*_GND_ in CP. High *ρ*_GND_ concentrations are observed at the stress-raiser, i.e. phase boundaries or notches, similar to results from single-crystal Ni during large thermal drop step.

### Sensitivity of ***C***_local_

As mentioned in the Methodology (Sect. "Hydride evolution"), the local hydrogen concentration can potentially reach up to 1000–3000 wppm near stress raisers such as crack tips (without liner) consistent with the neutron diffraction or APT data. However, the choice of local hydrogen content (over a more global average measurement) is not yet definitively clear. The hydrogen concentration limit, *C*_local_, is the value of H concentration assigned to initiate α-Zr to hydride phase transformation in the model. In this section, the hydriding results are repeated for the polycrystalline hydride growth when this limit changes to *C*_local_ = 1000 wppm, differing from the former value of 3000 wppm.

**Figure 16:**
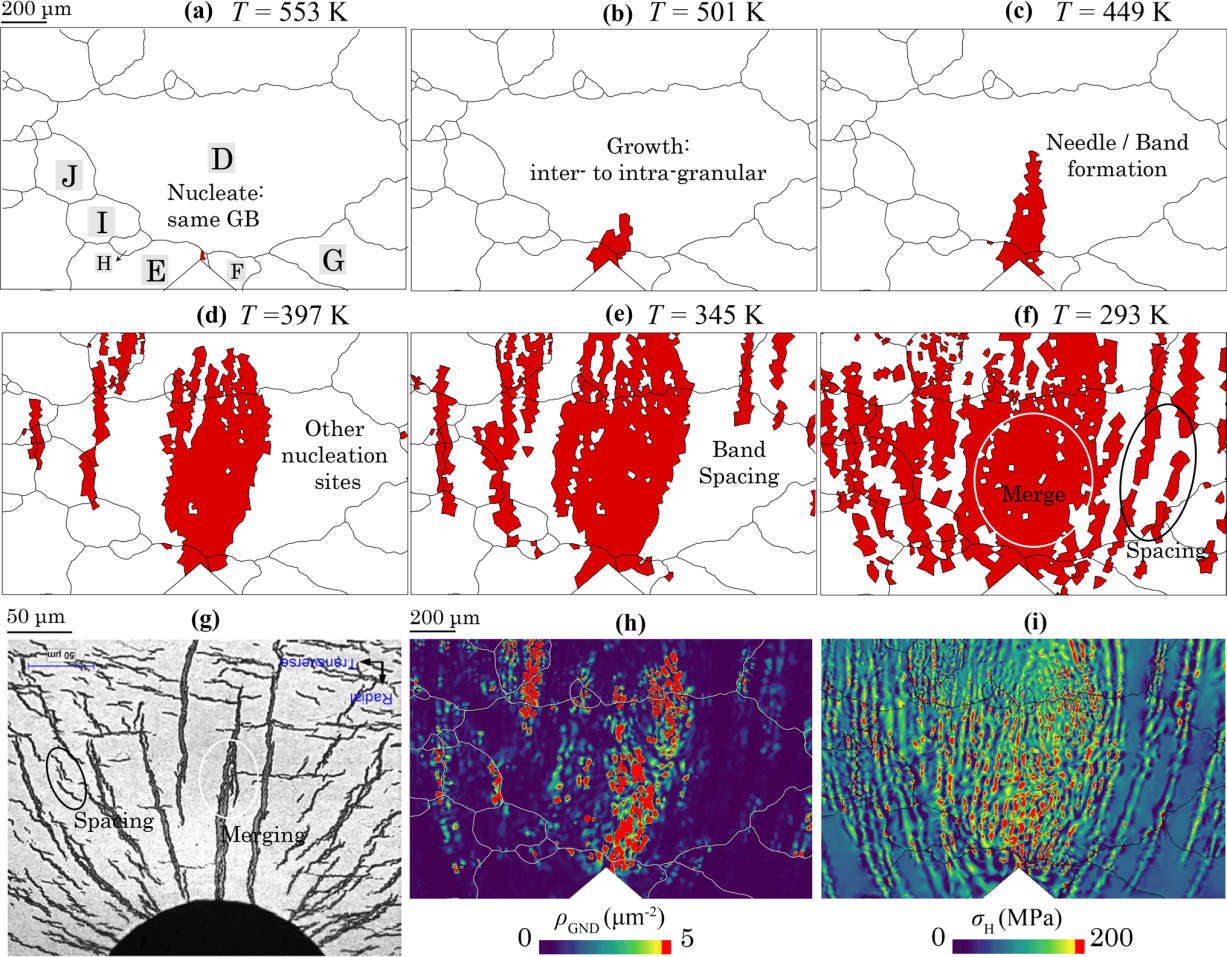
The effect of *C*_local_ when set to 1000 wppm, on the hydride evolution at *T* = **a** 553 K, **b** 501 K, **c** 449 K, **d** 397 K, **e** 345 K and **f** 293 K, comparing with **g** hydride distribution near notched zirconium alloy with a small grain size [67]. Band spacing hydrides are linked to the distribution of **h** *ρ*_GND_ and **i** *σ*_H_ at 293 K.

With the new, lower *C*_local_ limit, hydrides are predicted to start to form on the same grain boundary but closer to the notch tip, as shown in Fig. 16a. This is followed by the grain boundary and intragranular hydride growth near the D–E–F triple junction and notch tip in Fig. 16b. Band formation was observed at *t* = 12.5 h in Fig. 16c. This is consistent with experimental observation that showed the preferential growth of needle-shaped intragranular hydrides [29]. The needle-shaped hydride keeps growing axially in Fig. 16d, the mechanism for which is consistent with the study of Tondro et al. [46] showing higher hydrogen content at the needle tip than that at the sides due to tensile hydrostatic stress *σ*_H_ at the tip, whereas compressive *σ*_H_ develops at the sides. Other nucleation sites of hydrides also start to occur at *T* = 397 K in Fig. 16d.

The band spacing occurs between the needle-shaped hydrides growing from other nucleation sites in Fig. 16e. Some spaces are retained between hydride bands, whereas others merge as temperature reduces, as shown in Fig. 16f. The overall radially oriented hydride growth pattern follows the hydrostatic stress gradient around the notch tip, similar to the hydride growth pattern from Cui et al. in a notched specimen [67], as shown in Fig. 16g which also shows the hydride spacing, merging and shape of the stress field. It is noted that the grain boundaries are much denser in Cui et al. [67] since its grain size is ~ 10 µm compared to ~ 300 to 600 µm in current case, which means extensively more potential hydride nucleation sites since grain boundaries favours hydride formation. The hydride growth pattern mimics the case in Cui et al. due to more hydride nucleation sites under *C*_local_ = 1000 wppm. Besides, lowering *C*_local_ results in a higher rate for misfit strain incorporation, which is an analogue to higher cooling rate, consistent with more substantial hydride growth at high cooling rates from experimental observations [13]. Furthermore, the vast and quick hydride nucleation leads to the quicker hardening of the local material, which triggers stress redistribution and more aggressive hydride growth driven by local stress gradient.

The merging and spacing of hydrides in grain D are related to the distributions of *ρ*_GND_ and *σ*_H_ remaining inside the hydrides in Fig. 16h and i, respectively. Hot spots of *ρ*_GND_ and *σ*_H_ are linked to the hydride clusters in grain D with *c*-axis perpendicular to the observation plane, similar to former observations in the experiments [21]. The occurrence of hydride band spacing is explained by three reasons: (i) during hydride nucleation, low or compressive hydrostatic stress *σ*_H_ and high *ρ*_GND_ retained within individual hydrides caused by the volumetric misfit strain; (ii) distant nucleation sites lead to the separate *ρ*_GND_ and *σ*_H_ localisation bands, causing the band separation; (iii) during the formation of needle-shaped hydrides, high tensile *σ*_H_ exists at the tip of the needle hydrides whereas compressive *σ*_H_ at their sides. The side compressive *σ*_H_ pushes the hydrogen away from the side to the needle hydride tip, which leaves the space between neighbouring hydrides even when close to each other.

Regarding the importance of GND density, when a hydride needle grows in the model, *ρ*_GND_ increases during precipitation (resulting from slip and slip gradients within the hydride) and remains within the prior hydrided region during hydride dissolution. This is consistent with the dislocation structure formation and residual dislocations observed in TEM [17, 18]. As shown in a former study, the GND density and its distribution have a significant impact on localisation of hydrostatic stress |*E*_int_| and corresponding hydrogen near hydrides. If the hydride were to be assigned only elastic behaviour, the hydrostatic stress localisation would be relieved with the hydride growth, eventually leading to merging of multiple hydride bands thus inhibiting the formation of spaces between the needles.

### Effect of OR2

Another orientation relationship for zirconium alloy was reported to be OR2, which is $\left\{ {0001} \right\}_{{\upalpha }} \left| {\left| {\left\{ {001} \right\}_{{\updelta }} \left\langle {11\overline{2}0} \right\rangle_{{\upalpha }} } \right|} \right|\left\langle {110} \right\rangle_{{\updelta }}$ [12]. This was commonly observed mostly in zirconium hydride blister. Generally, where the total hydride concentration drops below 20%, less than 1% of the hydride by area is OR2 [12]. In the general in-service case, the hydride area fraction is mostly less than 20%. Nevertheless, it is useful when considering OR2 inside zirconium hydride blister, especially at the centre of the blister when local hydrogen concentration maximises. Comparing to OR1, the hydride formation follows the {001} plane instead of the {111} plane. This leads to 3 hydride variants, and the hydride forms a pattern like that of a Widmanstätten microstructure [12]. However, not many studies have been focused on the simulation of hydride formation for OR2, mostly due to its rare appearance, which only exists within zirconium alloy of high bulk hydrogen concentration (over 600 wppm in [12]).

In our methodology proposed, after the determination of the volumetric misfit strain and the elastic dipole tensor for OR2, the same logic could be followed to include hydride formation for OR2. The foreseeable question is that it would the local hydrogen concentration limit needs to determine for OR2 hydride formation. The OR2 delta hydride packet primarily existed in the blister hydride, where almost all α Zr turned into δ hydride. In the zirconium hydride blister, the OR1 is dominant with less than 20% of OR2 existence. Meanwhile, the area percentage of OR2 is higher at higher local hydrogen concentration. Therefore, it requires a thorough study on local hydrogen concentration from both experimental and simulative perspectives to determine the local hydrogen centration limit for OR2 hydride formation, ideally using high-resolution neutron radiography or even atom probe tomography. Furthermore, a variant selection methodology needs to be proposed in comparison with the experimentally observed microstructure of zirconium hydride blister. Nevertheless, it is predictable that OR2 hydride has higher misfit strain, and its formation requires higher interaction energy to balance the chemo-mechanical potential. Compared with OR2 hydride, the energy favourable OR1 will predominantly form in most of the hydride. From microstructure analysis [12], it is argued that only when local grain has preferable orientation for OR2 hydride with local high hydrogen concentration, such that the OR2 would form in alpha-Zr grain. In the meantime, the local high hydrostatic stresses would be mainly caused by the OR1 hydrides, i.e. the phase boundaries between parent α Zr grains and prior-formed OR1 hydride.

## Conclusion

In this study, hydride precipitation and dissolution are modelled and analysed for HCP polycrystalline material. The precipitation process is controlled by the external thermo-mechanical load, microstructure, crystal-level properties and hydride-matrix interactions. A DFT-informed CPFE method has been developed for hydride precipitation to understand the microstructure-sensitive hydride nucleation and growth in zirconium alloys. The mechanism of crystallography-sensitive hydride precipitation was studied in a contrived tri-crystal configuration with thermal or thermo-mechanical load. The micromechanical mechanisms were assessed during the precipitation process driven by the equilibrium between atomic interaction energy and chemical potential. Subsequently, polycrystalline hydride growth was modelled under industry-relevant thermo-mechanical loading in a large blocky α grains, showing consistent results with the experimental observations. The main conclusions are:

1. Planar hydride growth parallel to the basal planes followed by stacking growth is observed under thermal cooling conditions.
2. Hydrides prefer to grow within ‘hard’ grains, badly orientated for slip but with high hydrostatic stress, whereas ‘soft’ grains are not favoured, under thermo-mechanical load.
3. High absolute interaction energy |*E*_int_| indicates preferential hydride nucleation and growth sites in the polycrystal system.
4. Hydrogen content in a polycrystalline microstructure redistributes due to hydride growth and corresponding redistribution of |*E*_int_| and hydrostatic stress *σ*_H_.
5. CP predicted hydride growth pattern are consistent with the post-intergranular hydrides in experimental SEM observations for a blocky grain microstructure.
6. Deformation twin incorporation into the model induces hydride growth along twin layer due to localised hydrogen concentration at twin-matrix boundaries also confirmed by experimental observations.
7. A pre-thermal cooling process is essential to predict polycrystalline hydride precipitation due to GND accumulation from the temperature drop.
8. Lowering hydrogen content limit for hydride precipitation leads to claw-shaped hydride formation due to local stress gradient near notch tip, similar to experimental observations in small grain-sized microstructures.

## Figures and Tables

**Figure 17:**
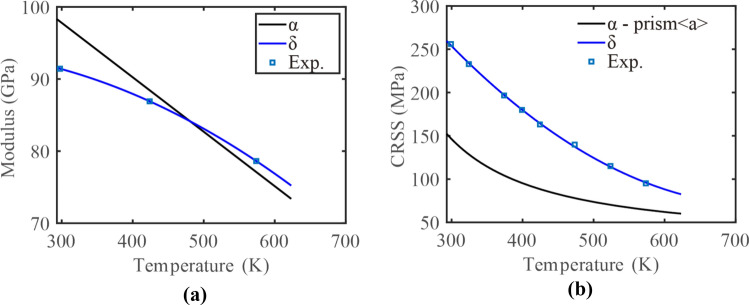
Thermal properties comparison between primary α, δ hydride and experimental data for hydride on **a** equivalent elastic modulus and **b** CRSS.

**Figure 18:**
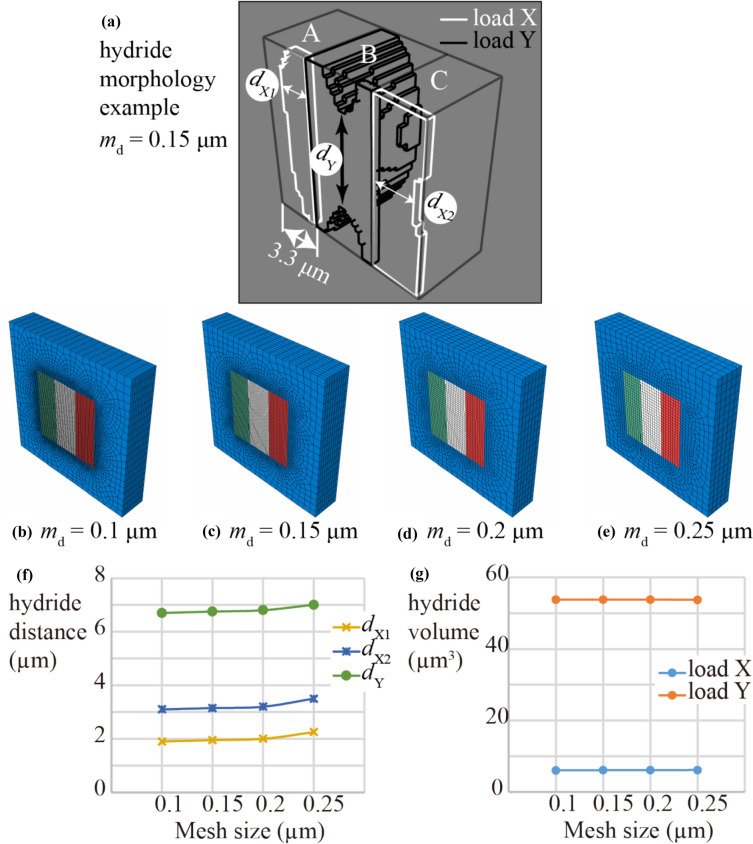
Mesh sensitivity study set-up includes **a** the hydride precipitation morphology under *m*_d_ = 0.15 µm (shown in Sect. "tri-crystal") to show the hydride morphological distances, i.e. *d*_X1_, *d*_X2_ and *d*_Y_, and **b**–**e** the four meshes applied in the simulations. **f** and **g** show the minor mesh size sensitivity of simulated hydride morphologies for both hydride distance and volume.

**Figure 19:**
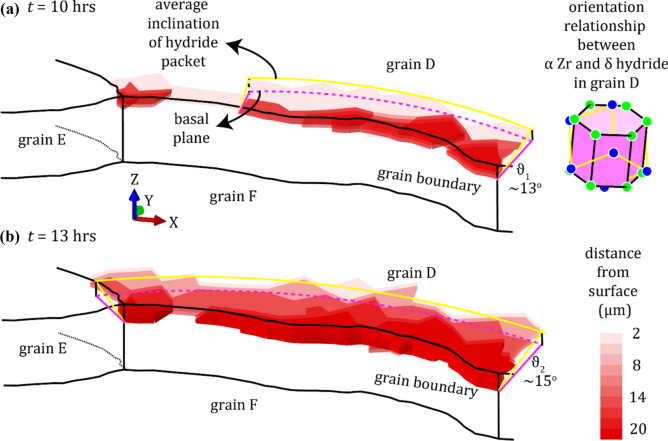
Hydride formation near D–F grain boundary at **a** *t* = 10 h and **b** *t* = 13 h to clearly show the 3D hydride morphology and orientation relationship with basal plane, which is consistent with that in tri-crystal case.

**Figure 20:**
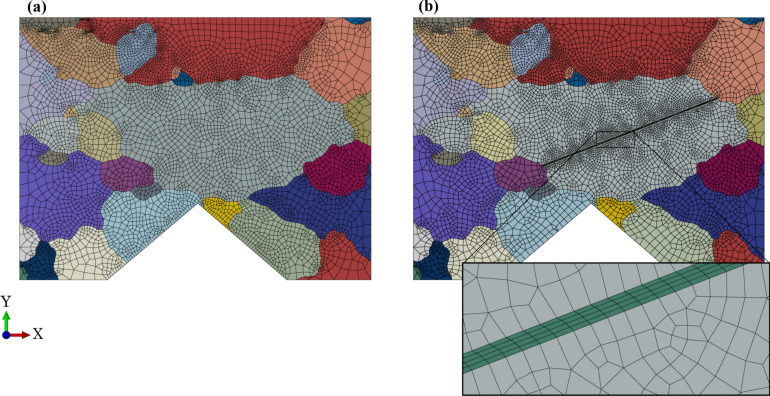
Mesh inside ROI for **a** original and **b** twin-embedded polycrystalline models.

**Figure 21:**
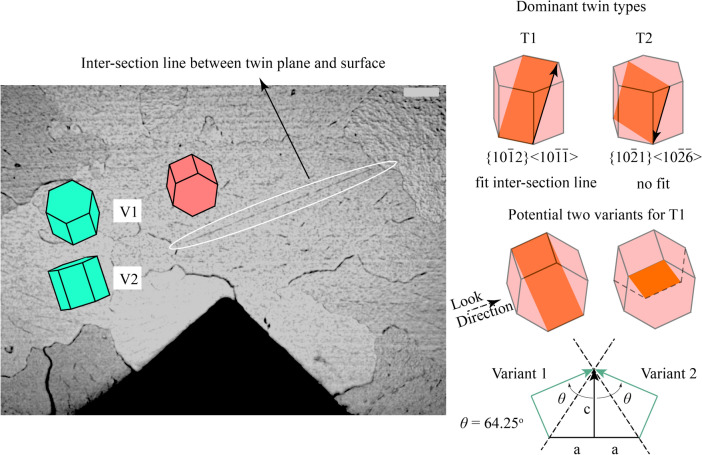
Twin identification satisfying the morphological direction of hydride-decorated twin observed from SEM.

**Figure 22:**
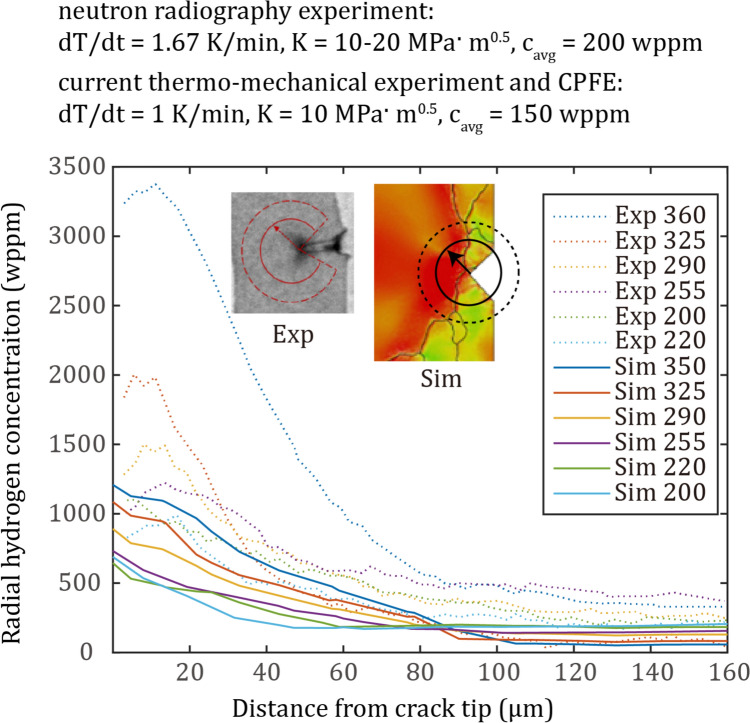
Comparison of hydrogen concentration near crack tip between the experimental results from neutron radiography and the CPFE results from current experiment.

## Data Availability

Data and code available under suitable request.

## Appendix A: Thermo-mechanical single-crystal properties of hydride and matrix

The crystal-level properties of matrix and hydrides were extracted from former studies [5, 68] and are shown in Tables

**Table 1:** Elasto-plastic material properties for prismatic slip in a Zr

Parameters		Unit	Prismatic <a> slip
Elastic	*E*_1_(*T*)	MPa	− 75.5 T + 120441.1
	*E*_3_(*T*)	MPa	− 32.7 T + 132862.1
	*G*_13_(*T*)	MPa	− 23.3 T + 38836.7
	*ν*_12_(*T*)		3.4 × 10^−4^* T* + 0.3002
	*ν*_13_(*T*)		− 9.1 × 10^−5^* T* + 0.2642
Plastic	*ρ*_m_	μm^−2^	0.01
	*ν*_*d*_	Hz	1.0 × 10^11^
	*b*^*κ*^	μm	3.2 × 10^–4^
	*k*	J K^−1^	1.38 × 10^–23^
	Δ*V*	μm^3^	20.93 b^3^
	${\tau }_{c,0}^{\kappa }\left(T\right)$	MPa	26.3 exp (514.7/T)
	Δ*F*	J	5.13 × 10^–20^
Thermal	*α*_*a*_	K^−1^	1.01 × 10^–5^
	*α*_*c*_	K^−1^	5.25 × 10^–6^

^1^ and

**Table 2:** Elasto-plastic material properties for δ hydride

Parameters		Unit	Slip system $\left\{111\right\}\langle \overline{1 }10\rangle$
Elastic	*E*(*T*)	GPa	− 7.0 × 10^−5^*T*^2^ + 0.015* T* + 93.24 [5]
	*ν*(*T*)		− 1.2 × 10^−5^* T* + 0.33 [71–73]
Plastic	*ρ*_m_	μm^−2^	0.01 [18]
	*ν*_*d*_	Hz	1.0 × 10^11^
	*b*^*κ*^	μm	4.78 × 10^–4^
	*k*	J·K^−1^	1.38 × 10^–23^
	Δ*V*	|*b*^*κ*^|^3^	0.3 [21]
	${\tau }_{c,0}^{\kappa }\left(T\right)$	MPa	9.4 × 10^−4^*T*^2^− 1.4* T* + 589.1 [5]
	Δ*F*	J	9.9 × 10^–20^ [21]
Thermal	*α*_*a*_	K^−1^	2.98 × 10^–6^ [74]

2. The thermal equivalent elastic modulus and CRSS of FCC δ hydride were derived and compared with the ones of α-Zr in Fig. 17. It should be noted that the CRSS ratio between prism <a>, basal <a> and pyramidal <c+a> slip systems are kept constant of 1:1.333:5.0 [69]. This might not be true as closer CRSS ratio between <a> and <c+a> slip systems at higher temperature was found in HCP titanium alloys [70].

## Appendix B: Computation of hydrogen concentration based on interaction energy

The computation starts with chemo-mechanical potential, which is a function of local Cauchy stress ***σ***, Green–Lagrange strain ***ε***, temperature *T*, and hydrogen concentration *c*,

9 $$F\left( {{\varvec{\sigma}},{\varvec{\varepsilon}},T,c} \right) = \mu \left( {T,c} \right) + E_{{{\text{int}}}} \left( {{\varvec{\sigma}},{\varvec{\varepsilon}}} \right)$$

where $\mu$ is the chemical potential based on temperature and hydrogen concentration,

10 $$\mu \left( {T,c} \right) = \mu_{0} + {\text{k}}T\ln \left( {\varphi c} \right);$$

*μ*_0_ is the reference potential of zirconium solvent, and k is the Boltzmann constant. *E*_int_ is the interaction energy defined as,

11 $$E_{{{\text{int}}}} \left( {{\varvec{\sigma}},{\varvec{\varepsilon}}} \right) = - \left( {E_{{\text{H}}} + E_{{\text{C}}} } \right) = - \left( {{\varvec{\varOmega}}:\left( {{\varvec{\varepsilon}}^{{\text{e}}} } \right)^{{\text{C}}} + {\varvec{\sigma}}^{{\text{C}}} :\left( {{\varvec{S}}_{{{\text{ZrH}}}} :{\varvec{\varOmega}}} \right)} \right)$$

In Fig. 3a, EH is the energy part due to H atom-induced lattice distortion exerted on neighbouring Zr lattice cells with elastic strain $\left( {{\varvec{\varepsilon}}^{e} } \right)^{C}$, and EC is the energy part from neighbouring stress state ${\varvec{\sigma}}^{C}$ applying on the lattice embedded with H atom. $S_{{{\text{ZrH}}}}$ is the compliance matrix of δ hydride from the DFT work [71]. ${\varvec{\varOmega}}\, = \,{\varvec{R}}^{T} \cdot{\varvec{\varOmega}}_{{0}} \cdot {\varvec{R}}$ is the atomic scale anisotropic elastic dipole tensor, which describes the work done by an H atom when located in its favourable tetrahedral site inside the HCP crystal. **R** is the grain orientation matrix from EBSD scanning. ***Ω***_0_ is the elastic dipole tensor at the initial configuration, computed from ab initio DFT methods [75], showing a larger value for the c-axis for occupying H atoms.

12 $$\left[ {{\varvec{\varOmega}}_{0} } \right] = \left[ {\begin{array}{*{20}c} {1.74 \pm 0.12} & 0 & 0 \\ 0 & {1.74 \pm 0.12} & 0 \\ 0 & 0 & {2.36 \pm 0.18} \\ \end{array} } \right]{\text{eV}}$$

Minimising chemo-mechanical potential, min. $[F({\varvec{\sigma}},{\varvec{\varepsilon}},T,c)]$, leads to the scale-bridging hydrogen concentration *c*,

13 $$c\left( {{\varvec{\sigma}},{\varvec{\varepsilon}},T} \right) = c_{0} \exp \left[ { - \frac{{E_{{{\text{int}}}} \left( {{\varvec{\sigma}},{\varvec{\varepsilon}}} \right)}}{{{\text{k}}T}}} \right]$$

and *c*_0_ is the initial bulk hydrogen concentration. A homogenisation method was introduced to obtain the stabilising hydrogen distribution [33].

## Appendix C: Mesh sensitivity

Mesh sensitivity study was conducted as shown in Fig. 20 to investigate the simulated hydride morphology under suitable mesh size, denoted by *m*_d_. As an example, the hydride morphology is shown for mesh size *m*_d_ = 0.15 µm in Fig 20a, which include 2 cases for both loading conditions along X and Y axes. Here, *d*_X1_ and *d*_X2_ represent the hydride morphological distance along the hydride precipitation direction (shown in Sect. "Tri-crystal"), whereas the *d*_Y_ represents the morphological distance for hydride merging. The morphological distances *d*_X1_, *d*_X2_ and *d*_Y_, as well as the volume of the hydride are investigated for simulated hydride precipitation under different mesh sizes, including *m*_d_ = 0.1, 0.15, 0.2 and 0.25 µm, as shown in Fig 20b–e. It is demonstrated in Fig 20f and g that the hydride morphological distances and hydride volume barely change with the mesh size, which means the hydride precipitation simulation under current mesh size of 0.15 µm is reasonable and reach the convergence for mesh sensitivity in finite element method. The hydride distance is slightly higher in the case of *m*_d_ = 0.25 µm, because the hydride precipitation morphology could not reach lower length scale due to the constraint of the large mesh size, resulting in the slight change of precipitated hydride morphology (Fig. 18).

## Appendix D: 3D hydride growth near grain boundary D–F

See Fig. 19.

## Appendix E: Polycrystalline mesh and twin identification

Polycrystalline meshes for ROI in Fig. 5b and twin modelling in Fig. 15 are shown in Fig. 18a and b, respectively. Nearly the same mesh size is applied in the untwined region for the two cases, whereas 4 element-thickness was applied in the twinned region in Fig. 20b to explicitly model the hydride growth inside twin.

In Fig. 21, dominant twin types are T1 and T2 twins under thermal cooling conditions where only T1 satisfies the morphological direction of the hydrided twin, i.e. the inter-section line between twin plane and surface. For the 2 crystallographic variants of twin, both of them were incorporated in the CPFE model separately and V1 shows the best hydride growth match to the experimental observation (Fig. 20 and 21).

## Appendix F: Polycrystalline mesh and twin identification

Hydrogen concentrations near crack tip are analysed using radial integral [54], in comparison with the experimental data from neutron radiography. The simulation results show that higher temperatures lead to higher hydrogen concentration near the crack tip, which is consistent to the experimental trend. This is due to the softening of dislocation slip strength, the bulk material strength, as well as stiffness at high temperature condition. This leads to the higher hydrostatic lattice deformation at higher temperature under the same external stresses, allowing more hydrogen to localise near crack tip. However, the hydrogen concentration level is not compatible, mainly caused by the differing microstructure and the shape and size of the crack tips in these two studies. Interestingly, a redistribution of hydrogen was observed in the CPFE simulation. Since the bulk hydrogen content barely changes during thermo-mechanical load, the localisation of hydrogen near crack tip results in the lower hydrogen concentrations away from the crack. This is shown specifically for the temperature at 255 °C in the neutron radiography case. However, the theoretical trend shown in CPFE is not entirely clear in the neutron experiment, since relative measurement of hydrogen concentration seems hard to distinguish in the high transmission region under the current technique reported [54] (Fig. 22).

## Abbreviations

APT: Atom probe tomography
CP: Crystal plasticity
CPFE: Crystal plasticity finite element
CRSS: Critical resolved shear stress
DFT: Density functional theory
DHC: Delayed hydride cracking
EBSD: Electron backscatter diffraction
FE: Finite element
FCC: Face-centred cubic
GND: Geometrically necessary dislocation
H: Hydrogen
HRDIC: High resolution digital image correlation
HCP: Hexagonal close packed
IGF: Inert gas fusion
OR: Orientation relationship
ROI: Region of interest
RSS: Resolved shear stress
SED: Stored energy density
SEM: Scanning electron microscopy
SRS: Strain rate sensitivity
SSD: Statistically stored dislocation
TEM: Transmission electron microscopy
TSSD: Terminal solid solubility for dissolution
TSSP: Terminal solid solubility for precipitation
XRD: X-ray diffraction
Zr: Zirconium
